# Unraveling Nitrogen, Sulfur, and Carbon Metabolic Pathways and Microbial Community Transcriptional Responses to Substrate Deprivation and Toxicity Stresses in a Bioreactor Mimicking Anoxic Brackish Coastal Sediment Conditions

**DOI:** 10.3389/fmicb.2022.798906

**Published:** 2022-02-23

**Authors:** Paula Dalcin Martins, Maider J. Echeveste Medrano, Arslan Arshad, Julia M. Kurth, Heleen T. Ouboter, Huub J. M. Op den Camp, Mike S. M. Jetten, Cornelia U. Welte

**Affiliations:** Department of Microbiology, RIBES, Radboud University, Nijmegen, Netherlands

**Keywords:** anaerobic methane oxidation, anammox, sulfide oxidation, substrate deprivation, toxicity stress, nitric oxide, enrichment culture

## Abstract

Microbial communities are key drivers of carbon, sulfur, and nitrogen cycling in coastal ecosystems, where they are subjected to dynamic shifts in substrate availability and exposure to toxic compounds. However, how these shifts affect microbial interactions and function is poorly understood. Unraveling such microbial community responses is key to understand their environmental distribution and resilience under current and future disturbances. Here, we used metagenomics and metatranscriptomics to investigate microbial community structure and transcriptional responses to prolonged ammonium deprivation, and sulfide and nitric oxide toxicity stresses in a controlled bioreactor system mimicking coastal sediment conditions. *Ca. Nitrobium versatile*, identified in this study as a sulfide-oxidizing denitrifier, became a rare community member upon ammonium removal. The ANaerobic Methanotroph (ANME) *Ca. Methanoperedens nitroreducens* showed remarkable resilience to both experimental conditions, dominating transcriptional activity of dissimilatory nitrate reduction to ammonium (DNRA). During the ammonium removal experiment, increased DNRA was unable to sustain anaerobic ammonium oxidation (anammox) activity. After ammonium was reintroduced, a novel anaerobic bacterial methanotroph species that we have named *Ca. Methylomirabilis tolerans* outcompeted *Ca. Methylomirabilis lanthanidiphila*, while the anammox *Ca. Kuenenia stuttgartiensis* outcompeted *Ca. Scalindua rubra*. At the end of the sulfide and nitric oxide experiment, a gammaproteobacterium affiliated to the family *Thiohalobacteraceae* was enriched and dominated transcriptional activity of sulfide:quinone oxidoreductases. Our results indicate that some community members could be more resilient to the tested experimental conditions than others, and that some community functions such as methane and sulfur oxidation coupled to denitrification can remain stable despite large shifts in microbial community structure. Further studies on complex bioreactor enrichments are required to elucidate coastal ecosystem responses to future disturbances.

## Introduction

Microorganisms drive and link the biogeochemical cycles of carbon, nitrogen, and sulfur by a variety of redox reactions (Madsen, 2011). Anthropogenic nutrient inputs from land into the ocean constitute a major impact on marine ecosystems, altering seawater and sediment biogeochemistry, and leading to increased primary production that can result in toxic algal blooms and oxygen depletion (Wallenius et al., 2021). Such impacts, combined with ocean warming and consequent seawater stratification and deoxygenation, can further stimulate the production of methane and nitrous oxide, potent greenhouse gases, as well as sulfide and nitric oxide (NO), toxic products of sulfate reduction and denitrification (Van Helmond et al., 2020; Malone and Newton, 2020; Wells et al., 2020; Wallenius et al., 2021). In coastal sediments, ammonium and nitrate can be introduced *via* agricultural runoff, while sulfide, nitrogen oxides, methane, and ammonium are generated *in situ via* sulfate reduction, partial denitrification, methanogenesis, and organic matter decomposition, respectively (Egger et al., 2018). Therefore, characterizing microbial communities, interactions, and reactions performed by microorganisms that couple methane, nitrogen, and sulfur cycling is fundamental for understanding biogeochemical cycling and linked greenhouse gas emissions in dynamic coastal ecosystems impacted by anthropogenic activity.

Modeling efforts suggest links between future environmental changes, biogeochemical cycles, and ecosystem functions (Treseder et al., 2012; Nazaries et al., 2013). However, given that most microorganisms are widespread and functionally redundant, they are frequently treated as a “black box” in models—preventing the effective modeling of their reactions and responses. Additionally, recent efforts with laboratory cultures and engineered systems have provided insights about impacts of substrate availability changes on environmentally and economically relevant microbial communities (Chen et al., 2017; Saad et al., 2017; Caffrey et al., 2019; Delgado Vela et al., 2021; Deng et al., 2021; Nie et al., 2021). Yet, few studies have examined microbial community responses to prolonged periods of substrate scarcity or environmental stresses in controlled systems (Bürgmann et al., 2011; Shade et al., 2012). However, these are highly relevant disturbances in coastal ecosystems, where, for instance, nitrogen limitation is a major control on eutrophication (Howarth and Marino, 2006), and sulfide toxicity can lead to mortality of marine life (Grieshaber and Völkel, 1998). Such studies are needed to unravel key microbial interdependencies, competitive interactions, and functional shifts, as well as to comprehend their environmental distribution and resilience under current and future disturbances.

Carbon-, nitrogen-, and sulfur-cycling microbial communities harbor potential for biotechnological applications such as the improvement of wastewater treatment systems. For instance, Deng et al. (2021) proposed that sulfide-driven partial denitrification could be coupled to anaerobic ammonium oxidation (anammox) in future applications, given that rapid oxidation of sulfide to elemental sulfur can prevent toxicity and inhibition of anammox activity. On the other hand, sulfide addition in a controlled aerated bioreactor setting promoted undesirable production of nitrous oxide and nitrite *via* partial denitrification and dissimilatory nitrite reduction to ammonium (DNRA), respectively (Delgado Vela et al., 2021). These observations indicate that further studies are necessary to understand complex microbial community interactions and to evaluate how they can be best employed in biotechnological applications.

In this research project, we investigated transcriptional stress responses of a complex microbial community enriched in an anoxic bioreactor mimicking dynamic and brackish sediment conditions, where periodic ammonium deprivation, and sulfide and NO toxicity stresses, the chosen stressors in this study, might occur. The culture performed sulfide, ammonium, and methane oxidation at the expense of nitrate *via* sulfide-oxidizing denitrifiers, anammox bacteria, and nitrite/nitrate-dependent anaerobic methane oxidizers (Arshad et al., 2017). The aims of this study were (i) to understand the effect of periodic ammonium removal on DNRA as a source of ammonium for anammox activity, as well as on general microbial community structure and transcriptional activity, (ii) to enrich sulfide-oxidizing NO reducers (potentially represented by *Ca*. *Nitrobium versatile*) while characterizing microbial community structure and transcriptional responses to prolonged sulfide and NO toxicity stresses, and (iii) to unravel potential metabolic reactions utilized by key community members while pursuing the first two aims.

## Materials and Methods

### Bioreactor Operation Under Regular and Experimental Conditions

**FIGURE 1:**
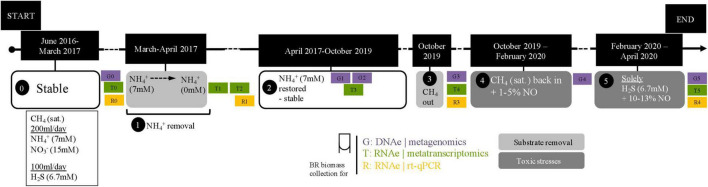
Timeline of experiments. Zero indicates when the bioreactor was stably maintained; 1, period of the ammonium removal experiment; 2, after the ammonium removal experiment and restabilization period; 3, reactor moved into a fume hood in preparation for the next experiment; 4, preparatory period for the nitric oxide and sulfide toxicity stresses experiment; 5, nitric oxide and sulfide toxicity stresses experiment. Samples for metagenomics (G0–G5), metatranscriptomics (T0–T5), and RT-qPCR (R0–R1 and R3–R4) were collected as indicated across experimental time points and in between them. The dashed line in the ammonium removal experiment indicates that removal was conducted stepwise (in mM: 7 → 4.5 → 3.5 → 2.5 → 0). Exact dates and experiment durations are provided in Supplementary Table 1.

In this study, we used a previously described complex coculture (Arshad et al., 2017). Briefly, this coculture was created by combining biomass from a marine enrichment culture containing sulfide-oxidizing and anammox bacteria (Russ et al., 2014) together with a freshwater nitrate/nitrite-dependent anaerobic oxidation of methane (N-AOM) enrichment culture dominated by *Ca. Methanoperedens archaea* and *Ca. Methylomirabilis bacteria* (Ettwig et al., 2016). After approximately 1 year, the stable coculture was characterized for substrate consumption and community composition (Arshad et al., 2017). This same stable coculture was subjected to a 10-week substrate starvation (ammonium removal) experiment (Figure 1, box 1), and later, after regular operation conditions were reestablished, to a 7-week sulfide and nitric oxide stress experiment (Figure 1, box 5). Briefly, under regular operation, the reactor culture was kept under anoxic conditions and fed daily with 0.67 mmol of sulfide, 1.4 mmol of ammonium, 3 mmol of nitrate, and 488 mmol of methane. The medium contained a solution of salts, trace elements, minerals, and vitamins as previously described (Arshad et al., 2017), and the pH was kept at 7.1 (Figure 1, box 0 and 4). Under experimental operation, the following conditions were modified. During the ammonium removal experiment, the ammonium concentration in the medium was gradually decreased from 7 to 0 mM over 1 month (Figure 1, box 1). Two more weeks passed without ammonium addition to the reactor in order to ensure that no residual ammonium was present, and the first biomass sample for metatranscriptomic sequencing (T1) was collected and stored at −80°C. After 10 more weeks without ammonium, another biomass sample for metatranscriptomic sequencing was collected, (T2) and ammonium was fully reintroduced into the medium. For exact dates, see Supplementary Table 1.

In preparation for the next experiment, the reactor was moved into a laminar flow hood. Methane flow was stopped at the time of reactor moving and reintroduced after 15 days (Figure 1, box 3). Once regular operation conditions and substrate consumption were reestablished, the sulfide and NO toxicity stress experiment began. Initially, the NO concentration in the reactor headspace was gradually increased to 1–5% for 10 weeks so that the community could adapt (Figure 1, box 4). Then, NO concentrations were increased to ∼10–13%, and all substrates except sulfide were removed from the reactor, resulting in only sulfide, NO, and medium were provided to the reactor for the following 7 weeks (Figure 1, box 5). A biomass sample for metatranscriptomic sequencing was collected before any NO was added to the reactor (T4) and after the 17 weeks (T5). Originally, the experiment was planned to be conducted in 20 weeks, as during the 10 last weeks the reactor would be maintained under only sulfide and NO. However, the COVID-19 lockdown in the Netherlands resulted in restricted access to the laboratory, and the timing had to be changed from 20 to 17 weeks. Additional metatranscriptomics samples in between experiments were included in this study: T0, approximately 2 months before the ammonium removal experiment, and T3, 6 months after the ammonium removal experiment and 11 months before the sulfide and NO stress experiment (Figure 1 and Supplementary Table 1).

During regular and experimental conditions, the reactor was checked daily for general parameters including pH with an Applisens electrode (Applikon, Delft, Netherlands) and nitrate and nitrite concentrations with MQuantTM colorimetric test strips (Merck, Darmstadt, Germany). During experimental conditions, nitrate and nitrite were also measured with the Griess assay as previously described (Arshad et al., 2017), and ammonium was measured fluorometrically after reaction with 10% orthophthaldialdehyde as previously described (Taylor et al., 1974). NO in headspace samples was measured with a Sievers Nitric Oxide analyzer (NOA280i; GE Power Water & Process Technologies, Boulder, CO, United States), and sulfide in liquid samples was measured with the methylene blue assay (Moest, 1975) (HACH, Loveland, CO, United States). Sulfate was determined *via* the barium precipitation method using the Sulfate Assay Kit following the instructions of the manufacturer (Sigma-Aldrich, St. Louis, MI, United States). Sample measurements were carried out in technical triplicates.

### Nucleic Acid Extractions and Sequencing

Biomass samples for metagenomic sequencing were collected during regular and experimental periods (Figure 1) and stored at −20°C. All DNA extractions were performed using the DNAeasy Power Soil Kit (Qiagen, Hilden, Germany) following the instructions of the manufacturer with two modifications: bead beating was performed in a TissueLyser (Qiagen, Hilden, Germany) for 10 min, and autoclaved MilliQ water was used instead of the kit’s buffer in the last elution step. RNA extractions were initially performed (for T1 and T2) using the RiboPure Bacteria Kit (Thermo Fisher Scientific, Waltham, MA, United States) following the instructions of the manufacturer. During the stress experiment (T4 and T5), this method no longer resulted in sufficient extracted RNA for sequencing, potentially due to extensive extracellular polymeric substance production, and thus, the method was changed to RNeasy Power Soil Kit (Qiagen, Hilden, Germany), which was also used for two additional samples (T0 and T3). All RNA samples were treated with DNAase I at 37°C for 30 min from the RiboPure Bacteria Kit (Thermo Fisher Scientific, Waltham, MA, United States). RNA extractions were performed in three biological replicates. Both RNA extraction for RT-qPCR and metatranscriptomics were subjected to the same DNAase treatment. After this treatment, RT-qPCR RNA extractions were tested for DNA contamination. As explained below, RNA samples detected background DNA contamination, equivalent to CT values of RNA extractions of negative controls (water). In addition, we also performed PCR on RNA extractions for RT-qPCR and did not observe any PCR products, thus, validating the effectiveness of the DNAase treatment in trace DNA removal for all RNA extractions employed. DNA and RNA concentrations were measured with a Qubit 2.0 fluorometer using the dsDNA and RNA HS kits (Thermo Fisher Scientific, Waltham, MA, United States). DNA and RNA quality were determined using a NanoDrop Spectrophotometer ND-1000 (Isogen Life Science, Utrecht, Netherlands) and a Bioanalyzer 2100 (Agilent, Santa Clara, CA, United States), respectively.

Metagenomic libraries were prepared using the Nextera XT Kit (Illumina, San Diego, CA, United States) following the instructions of the manufacturer. Enzymatic tagmentation was performed with 1 ng of DNA, followed by the incorporation of indexed adapters and amplification of the library. Amplified DNA libraries were then purified, and their quality and concentration were determined as aforementioned. Libraries were sequenced on an Illumina MiSeq platform (San Diego, CA, United States) using the MiSeq Reagent Kit v3 (San Diego, CA, United States), generating 300-bp paired-end reads. RNA samples of T1 and T2 were rRNA-depleted with MICROBExpress Kit (Thermo Fisher Scientific, Waltham, MA, United States) and MEGAclear kit (Ambion, Life Technologies, Carlsbad, CA, United States). Subsequently, 0.1–4 μg of RNA from T1 to T3 were used to construct strand-specific RNA-Seq libraries. Non-rRNA in RNA-Seq libraries were enriched by selective priming during the first-strand cDNA synthesis reaction, as well as in the final library construction steps using TruSeq Stranded mRNA sample preparation guide (Illumina proprietary catalog RS-122-9004DOC). RNA from these samples was sequenced with an Illumina MiSeq platform (Illumina, San Diego, CA, United States), generating 151-bp single-end reads. Metatranscriptomic libraries of T0, T4, and T5 were constructed using TruSeq stranded mRNA library Kit (Illumina, San Diego, CA, United States). These RNA-Seq libraries were sequenced with an Illumina NovaSeq 6000 platform, generating 151 bp paired-end reads (San Diego, CA, United States). All metatranscriptomes were triplicate RNA extractions and sequencing.

### Metagenomic and Metatranscriptomic Analyses

Metagenomic data were analyzed as follows. Read quality was assessed with FASTQC v0.11.8 before and after quality trimming, adapter removal, and contaminant filtering, performed with BBDuk (BBTools v38.75). Trimmed reads were coassembled *de novo* using MEGAHIT v1.2.911 (Li et al., 2016) and mapped to assembled contigs using BBMap (BBTools v38.75) (Bushnell, 2016). Sequence mapping files were handled and converted using SAMtools v1.10. Contigs at least 1,000-bp long were used for binning with CONCOCT v1.1.0 (Alneberg et al., 2014), MaxBin2 v2.2.7 (Wu et al., 2015), and MetaBAT2 v2.1512 (Kang et al., 2019). Resulting metagenome-assembled genomes (MAGs) were dereplicated with DAS Tool v1.1.213 (Sieber et al., 2018) and taxonomically classified with GTDB-Tk v1.3.0 (Chaumeil et al., 2019) release 9514. MAG completeness and contamination was estimated with CheckM v1.1.2 (Parks et al., 2015).

Metagenome-assembled genomes were annotated with DRAM v1.0 (Shaffer et al., 2020) with default options, except min_contig_size at 1,000 bp, and genes of interest were searched in annotation files as well as *via* BLASTP and HMM analyses. Only high- and medium-quality MAGs (>50% complete and <10% contaminated) were included in genome-centric analyses, and the entire dataset (binned and unbinned contigs) was considered in gene-centric analyses. For phylogenetic trees, sequences were aligned with MUSCLE v3.8.31 (Edgar, 2004), alignment columns were stripped with trimAl v1.4.rev22 (Capella-Gutierrez et al., 2009) using the option -gappyout, and trees were built with FastTree v2.1.10 (Price et al., 2010) or UBCG v3.0 (Na et al., 2018) and visualized with iToL v6 (Letunic and Bork, 2021). For calculating average amino acid identity (AAI) between selected MAGs, genomes were gene-called with Prodigal v2.6.3 (Hyatt et al., 2010), and amino acid fasta files were used as input to the Kostas Lab online tool^1^ (Rodriguez-R and Konstantinidis, 2016). The Genome-to-Genome Distance Calculator v3.0 tool was used online^2^ (Meier-Kolthoff et al., 2013). Heat maps were constructed with the package gplot, function heatmap.2 on RStudio v1.3.959, R v4.0.4.

Metatranscriptomic reads were quality trimmed with Sickle v1.33 (Joshi and Fass, 2011) using the sickle se (single end) or pe (paired-end) options for Sanger sequencing (-t sanger). Trimmed transcripts were mapped against the annotated metagenome with Bowtie2 (Langmead and Salzberg, 2012) (bowtie2 -D 10 -R 2 -N 1 -L 22 -i S,0,2.50 -q -a -p 30), allowing only one mismatch. Index stats files were imported into RStudio to calculate transcripts per million (TPM) values according to the formula TPM = [number of reads/(gene length/10^3^)]/10^6^, which were used as unit of gene transcription. Aware of differing extraction and sequencing methods for T1 and T2, these two data points have only been used in separate analyses. TPM values were used for bubble plot generation with the packages ggplot on RStudio. All figures were layout edited using Adobe Illustrator CC v22.1.

### Reverse-Transcription Quantitative Polymerase Chain Reaction

Selected RNA samples were used for RT-qPCR (Figure 1) in order to confirm patterns emerging from metatranscriptomic analyses. Three primer pairs were selected to quantify transcription of genes of interest: hydrazine synthase subunit A, with *hzsA*-F (5′-WTCGGRTATCARTATGTAG-3′) and *hzsA*-R (5′-AAATGGYGAATCATARTGGC-3′), adapted from previously published primers (Harhangi et al., 2012); particulate methane monooxygenase subunit A, with *pmoA*-F (5′-SCGRGTRMAGCCSGGTGAGA-3′) and *pmoA*-R (5′-YGATGGYCCMGGYACMGAGT-3′), designed for this study; and methyl-coenzyme M reductase subunit A, with *mcrA*-F (5′-AAAGTGCGGAGCAGCAATCACC-3′) and *mcrA*-R (5′-TCGTCCCATTCCTGCTGCATTGC-3′) (Vaksmaa et al., 2017). Bacterial and archaeal 16S rRNA gene-targeting primers 16S-F (5′- AAACTYAAAKGAATTGRCGG′) and 16S-R (5′-ACGGGCGGTGWGTRC-3′) were used as housekeeping genes for data normalization, adapted from previously published primers (Engelbrektson et al., 2010). For each sample, 50 ng of RNA was used for the reverse transcription reaction using the RevertAid H Minus First Strand cDNA Synthesis Kit (Thermo Fisher Scientific, Waltham, MA, United States) following instructions of the manufacturer. Each qPCR reaction consisted of 12 μl of SYBR Green FastMix (QuantaBio, Beverly, MA, United States), 1 μl forward and 1 μl reverse primers (10 μM working solutions), 10 μl DEPC-treated water (Thermo Fisher Scientific, Waltham, MA, United States), and 1 μl of template single-stranded cDNA in a total volume of 25 μl. The qPCR program consisted of the following steps: initial denaturation (94°C for 5 min), denaturation (94°C for 30 s), annealing (temperature as below for 30 s), elongation (72°C for 30 s), and melting curve (50–95°C, 0.5°C increase per 5 s). Denaturation, annealing, and elongation steps were repeated for 40 cycles. Annealing temperatures for *hzsA*, *pmoA*, *mcrA*, and general 16S rRNA gene primers were 55, 62, 62, and 55°C, respectively. To check for DNA contamination, qPCR was performed directly on RNA samples. This confirmed that DNA contamination was neglectable, given that all CT values were above the threshold of 30 cycles. RT-qPCR units were expressed in 2^–ΔΔCT^ by subtracting, first, normalizer with target functional gene CT values, and second, R0 and R3 from R1 and R4 CT values, respectively. Replicates were discarded if CT values were above 30 cycles.

### Data Availability

Metagenomic trimmed reads, metatranscriptomic trimmed reads, metagenome-assembled genomes, and unbinned contigs have been deposited on NCBI under BioProject number PRJNA758578.

## Results

### Diverse Microbial Community Members Cycled Methane, Nitrate, Nitrite, Ammonium, and Sulfide

For over 1 year since the combination of the two parent enrichment cultures, the bioreactor ran under stable conditions (Figure 1, box 1; Arshad et al., 2017). During the ammonium removal experiment, the bioreactor received the same daily quantities of nitrate (3 mmol), methane (488 mmol), and sulfide (0.67 mmol) as previously described (Arshad et al., 2017). A comprehensive investigation of daily substrate consumption was carried out in the aforementioned study (corresponding to the zero, Figure 1, box 1), which showed that sulfide was completely consumed with a nitrate removal rate of 2.6 mmol per day. The residual nitrate amount fluctuated around 0.4 mmol/day, while no nitrite could be detected in the bioreactor (Arshad et al., 2017). In the present investigation, ammonium was removed from the medium (Figure 1, box 1), and nitrate, nitrite, and ammonium substrate consumption was monitored for a period of 12 days prior to metatranscriptomics biomass (T2) collection (10 weeks without ammonium). Nitrate had a residual concentration of 3 mM, while ammonium, nitrite, and sulfide concentrations remained below the detection limit. The overall nitrate and nitrite concentrations were consistent with the previously determined concentrations (Arshad et al., 2017).

During the sulfide and NO toxicity stress experiment (Figure 1, box 5), methane, nitrate, and ammonium were not provided to the reactor. Only sulfide and NO were added as substrates in order to enrich sulfide-oxidizing nitric oxide reducers. While sulfide concentrations remained below detection limit for the entire experiment, indicating continuous complete sulfide removal, nitrite accumulated to 870 μM during the second week of the bioreactor receiving only sulfide and NO, and remained between 100 and 400 μM during the remainder of the experiment. This indicated that the community removed residual nitrate (834 μM) *via* denitrification and DNRA for several weeks. Biomass decay was evident from ammonium concentrations (0–500 μM), the visual increase in reactor turbidity, and to a degree, change in granule color from red to black, until the end of the experiment.

**FIGURE 2:**
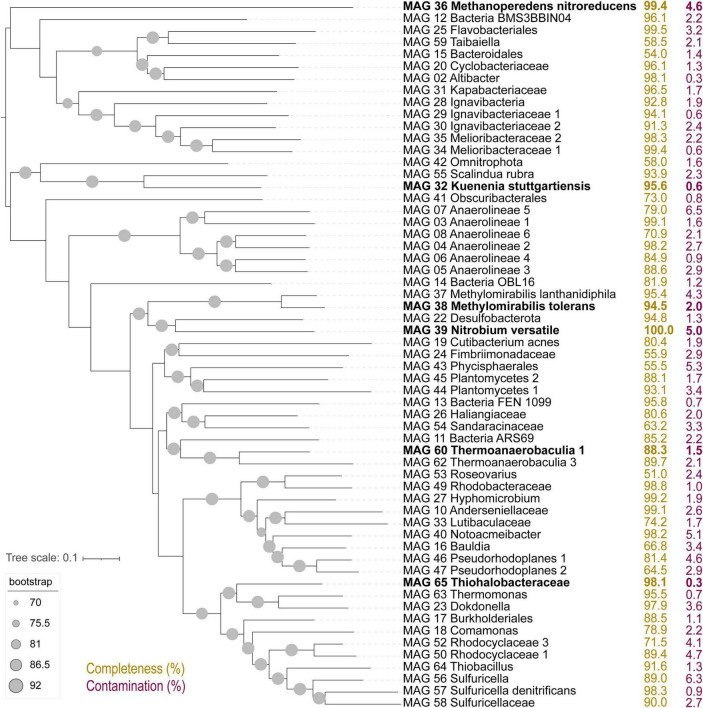
Up-to-date bacterial core gene (UBCG) tree of 92 concatenated genes extracted from high- and medium-quality metagenome-assembled genomes (MAGs) in this study. Genome completeness (in yellow) and contamination (in purple) values in percent are indicated to the right. MAGs in bold were selected from detailed characterization due to large shifts in abundance across experiments.

The composition of the bioreactor microbial community was investigated *via* a time series of metagenomic sequencing (Figure 1, G0–G5). In a previous study with this bioreactor, community members included several proteobacteria, *Ca. Kuenenia stuttgartiensis*, *Ca. Scalindua brodae*, *Ca. Methanoperedens nitroreducens*, *Ca. Methylomirabilis* species, and a novel bacterium within the *Nitrospirota* phylum, *Ca. Nitrobium versatile*, potentially linking sulfur and nitrogen cycling (Arshad et al., 2017; Zecchin et al., 2018; Umezawa et al., 2020, 2021). Coassembly of eight samples resulted in 30 high (>90% complete, <5% contaminated) and 29 medium (>50% complete, <10% contaminated) quality metagenome-assembled genomes (MAGs) (Figure 2). We will first introduce the most dominant members, and in the next section, we will discuss their change in abundance over time. The only archaeon detected in the bioreactor was *Ca. Methanoperedens nitroreducens* (MAG 36), a nitrate-reducing anaerobic methanotroph. Bacteria were much more diverse and represented by 5 MAGs affiliated to the candidate phyla (AABM5-125-24, ARS69, FEN-1099, GWC2-55-46, and OLB16), 2 to *Acidobacteriota*, 1 to *Actinobacteriota*, 1 to *Armatimonadota*, 11 to *Bacteroidota*, 6 to *Chloroflexota*, 1 to *Cyanobacteria*, 2 to *Methylomirabilota*, 2 to *Myxococcota*, 1 to *Nitrospirota*, 1 to *Omnitrophota*, 5 to *Planctomycetota*, and 20 to *Proteobacteria*.

**FIGURE 3:**
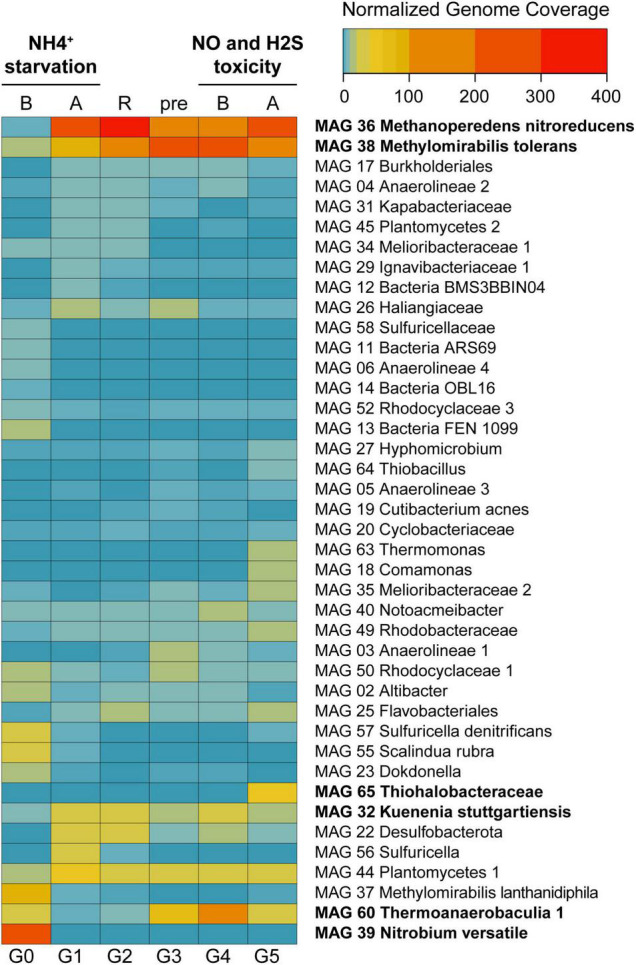
Heat map displaying normalized genome coverage for the most abundant MAGs across experimental conditions. Abbreviations are as follows: B, before; A, after; R, recovery; pre, preparatory phase. Experiments are indicated in bold on top. MAGs are ordered based on normalized genome coverage values and abundance change patterns. Between 91 and 95% of the reads were assembled and mapped to the MAGs across all experimental time points.

**FIGURE 4:**
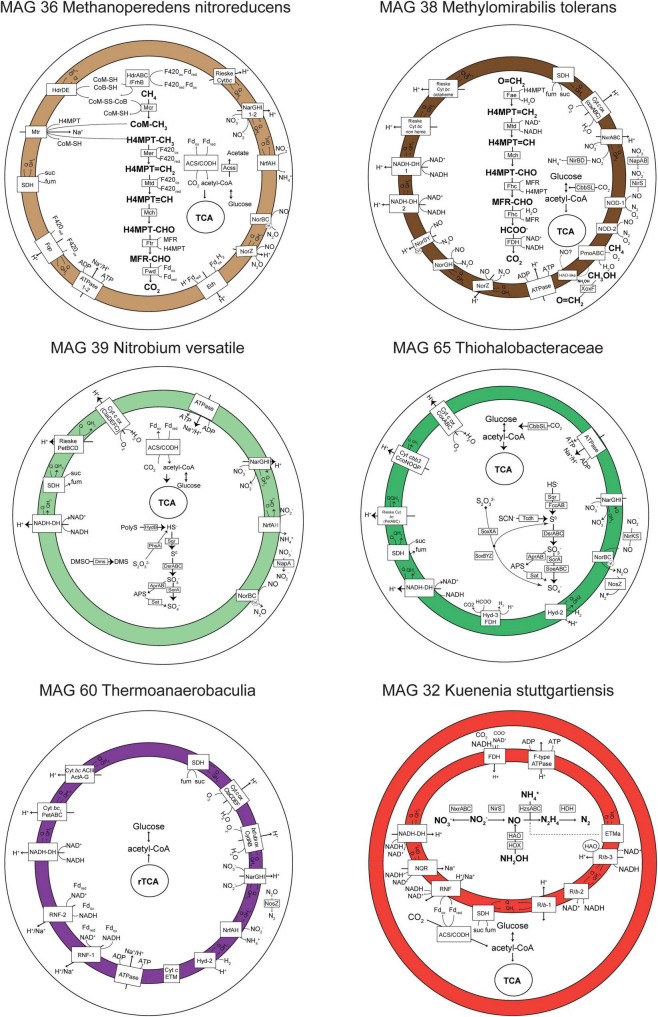
Metabolic reconstruction of six genomes of interest. Only genes with TPM > 0 in metatranscriptomic analyses are shown in selected time points in which the highest number of genes were transcribed (T5 for MAGs 36, 60, and 65, T4 for MAG 38, and T0 for MAGs 32 and 39). Gene abbreviations, loci, and annotations are provided in Supplementary Table 4. The gene *nrfH* was absent in MAG 39 *Ca. Nitrobium versatile* (in gray), but it was previously identified in the first MAG representing this organism (Arshad et al., 2017). In MAG 32 *Ca. Kuenenia stuttgartiensis*, the internal compartment represents the anammoxosome.

**FIGURE 5:**
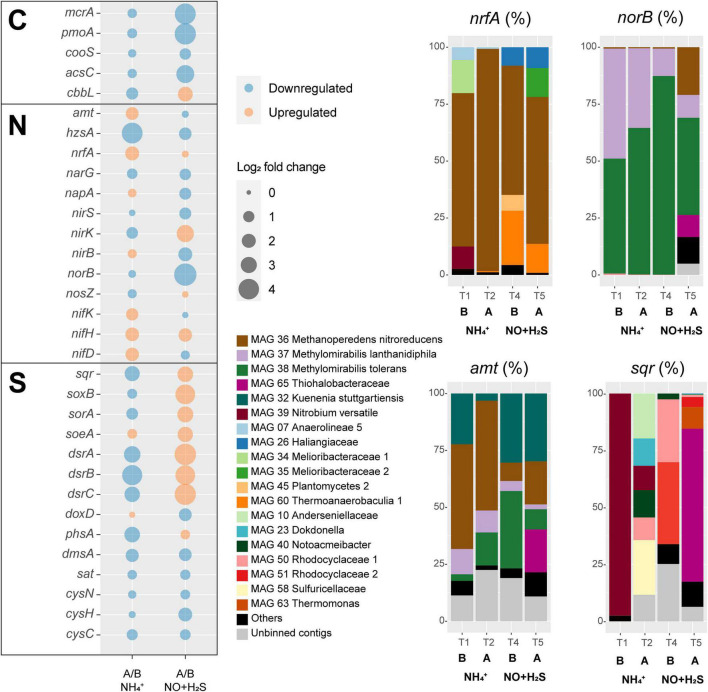
Transcriptional responses to ammonium removal and sulfide and NO toxicity experiments. Left: log2 fold change, as well as upregulation or downregulation of genes encoding proteins involved in carbon (C), sulfur (S), and nitrogen (N) cycling. In this analysis, all binned and unbinned contigs were analyzed together. Right: taxonomic contribution to selected gene transcripts of MAGs and unbinned contigs. Under *norB*, subunits of putative nitric oxide dismutases are also included. Abbreviations are as follows: B, before; A, after.

Two of the *Planctomycetota* MAGs were classified by GTDB-Tk as the anammox bacteria *Ca. Kuenenia stuttgartiensis* (MAG 32) and *Ca. Scalindua rubra* (MAG 55). The one *Nitrospirota* MAG 39 was *Ca. Nitrobium versatile*, which we further characterized in this study. The two MAGs affiliated to *Methylomirabilota* were classified by GTDB-Tk as *Ca. Methylomirabilis oxyfera* (MAG 37) and *Ca. Methylomirabilis sp002634395* (MAG 38). However, phylogenetic and average amino acid identity (AAI) analyses revealed that the first MAG represented a species of *Ca. Methylomirabilis lanthanidiphila* [100% AAI to the type genome (Versantvoort et al., 2018)], and the latter was a novel species sharing only 81% AAI to *Ca. Methylomirabilis limnetica* (Supplementary Figures 1, 2). The genome-to-genome distance between MAG 38 and *Ca. M. limnetica* ranged from 0.15 to 0.59, with a probability of DNA–DNA hybridization >70% ranging from 0 to 0.08%, supporting AAI results. We have named this novel species *Ca. Methylomirabilis tolerans* due to its persistence over the sulfide and NO stress experiment (Figure 3). Genome analyses indicated that *Ca. Methylomirabilis tolerans* had a similar metabolic potential for NO dismutation coupled to methane oxidation *via* intracellular oxygen generation as previously described, sharing several other characteristics with *Ca. Methylomirabilis lanthanidiphila* (Table 1). Finally, several previously identified and novel putative sulfide oxidizers were identified based on key gene analyses (Figures 4, 5): MAG 58 *Sulfuricellaceae*, MAG 10 *Anderseniellaceae*, MAG 23 *Dokdonella*, MAG 40 *Notoacmeibacter*, MAG 50 *Rhodocyclaceae 1*, MAG 52 *Rhodocyclaceae 3*, MAG 57 *Sulfuricella denitrificans*, MAG 56 *Sulfuricella*, MAG 64 *Thiobacillus*, MAG 65 *Thiohalobacteraceae*, and MAG 63 *Thermomonas*.

**TABLE 1:** Comparison of functional genes and pathways in selected *Ca. Methylomirabilis* species from this and other studies.

Reaction	*Ca*. *M. oxyfera* (Versantvoort et al., 2018)	*Ca*. *M. lanthanidiphila* (Versantvoort et al., 2019)	*Ca*. *M. tolerans* (this study)	*Ca*. *M. limnetica* (Graf et al., 2018)
**Carbon metabolism**				
Carbon fixation	Calvin cycle	Calvin cycle	Calvin cycle	Calvin cycle
Methane oxidation	pMMO	pMMO	pMMO	pMMO
Methanol oxidation	MxaFI, XoxF	XoxF	XoxF	XoxF
Formaldehyde oxidation	H_4_F, H_4_MPT	H_4_F, H_4_MPT	H_4_F, H_4_MPT	H_4_F, H_4_MPT
Formate oxidation	FDH	FDH	FDH	FDH
**Nitrogen metabolism**				
Nitrate reduction/nitrite oxidation	Nxr, Nap	Nxr, Nap	Nxr, Nap	Nap
Nitrite reduction to nitric oxide	NirS	NirS	NirS	NirS
Nitrite reduction to ammonium	NirBD fusion	NirBD fusion	NirBD fusion	NI
Nitric oxide dismutation	NOD	NOD	NOD	NOD
Nitric oxide reduction	qNOR, sNOR, gNOR	qNOR, sNOR, gNOR	qNOR, sNOR, gNOR	qNOR
Nitrous oxide reduction	NI	NI	NI	NI
Oxygen reduction	COX	COX	COX	COX
Hydroxylamine oxidation	Hao-like	Hao-like	Hao-like	–
**Others**				
*bc*_1_ complex	Canonical	Min. 2 non-canonical	Min. 2 non-canonical	Min. 2 non-canonical
Gas vesicle	NI	NI	NI	Yes (*gvpA* and associated proteins)

*pMMO, particulate methane monooxygenase; MxaFI, calcium-dependent methanol dehydrogenase; XoxF, lanthanide-dependent methanol dehydrogenase; H_4_F, tetrahydrofolate pathway; H_4_MPT, tetrahydromethanopterin pathway; FDH, formate dehydrogenase; Nxr, membrane-bound nitrate reductase/nitrite oxidoreductase phylogenetically closest to nitrite-oxidizing enzyme sequences; Nap, periplasmic nitrate reductase; NirS, cytochrome cd_1_-containing nitrite reductase; NirBD, cytoplasmic ammonium-forming nitrite reductase; NOD, nitric oxide dismutase; qNOR, quinone-oxidizing electrogenic nitric oxide reductase; sNOR, cytochrome c-oxidizing electrogenic nitric oxide reductase; gNOR, quinone-oxidizing non-electrogenic nitric oxide reductase; COX, cytochrome c oxidase complex; Hao, hydroxylamine oxidoreductase; NI, not identified.*

### Metagenome-Assembled Genome Coverages Revealed Shifts in Microbial Community Structure During Experimental Conditions

Normalized genome coverage for each MAG was used as a proxy for organism abundances across time points. Under regular operation conditions (Figure 1, G0), the most abundant microorganism in the reactor was MAG 39 *Nitrobium versatile*, with a genome coverage of approximately 245× (Figure 3). MAG 37 *Methylomirabilis lanthanidiphila* had normalized genome coverage of 89×, MAG 60 *Thermoanaerobaculia 1* of 26×, MAG 57 *Sulfuricella denitrificans* of 26×, and MAG 55 *Scalindua rubra* of 25×. However, large shifts in microbial community structure occurred after ammonium was removed from the medium and subsequently reintroduced (G1). MAG 36 *Methanoperedens nitroreducens*, which previously had a normalized genome coverage of 5×, increased to 278×, and MAG 38 *Methylomirabilis tolerans*, which had a coverage of 19× before, increased to 84×, while MAG 37 *Methylomirabilis lanthanidiphila* decreased to 4×. Other abundant genomes at G1 were MAG 44 *Planctomycetes 1* (50×), MAG 22 *Desulfobacterota* (39×), MAG 32 *Kuenenia stuttgartiensis* (37×), and MAG 56 *Sulfuricella* (37×). These genomes remained the most abundant in the bioreactor during the recovery period (G2), with MAG 36 *Methanoperedens nitroreducens* reaching 366× coverage, and MAG 38 *Methylomirabilis tolerans*, 110×, indicating that the community reached another but distinct stable structure. During the preparatory phase for the sulfide and NO toxicity experiment (G3), MAG 38 *Methylomirabilis tolerans* became dominant (292×), followed by MAG 36 *Methanoperedens nitroreducens* (126×), MAG 60 *Thermoanaerobaculia 1* (71×), MAG 44 *Planctomycetes 1* (33×), and MAG 32 *Kuenenia stuttgartiensis* (18×). These MAGs remained, in this order, the most abundant genomes in the reactor until the sulfide and NO toxicity experiment started (G4). Finally, at the end of this experiment (G5), again, shifts in community structure were detected: MAG 36 *Methanoperedens nitroreducens* returned as the dominant genome (207×), followed by MAG 38 *Methylomirabilis tolerans* (198×), MAG 65 *Thiohalobacteraceae* (52×), MAG 44 *Planctomycetes 1* (36×), MAG 60 *Thermoanaerobaculia 1* (29×), and MAG 32 *Kuenenia stuttgartiensis* (19×).

### Metatranscriptomic Analyses Reveal Active Metabolic Pathways in Key Microbial Community Members Cycling Methane, Nitrogen, and Sulfur

To investigate transcriptional responses of the bioreactor microbial community to experimental conditions and to unravel microbial pathways likely utilized for metabolic activity in the reactor, we conducted a time series of metatranscriptomic sequencing. With these data, we calculated transcript per million (TPM) values as units of gene transcription. We had particular interest in microorganisms that achieved highest abundances or persisted across experimental time points, and selected several key species for in-depth metabolic reconstruction: two key putative sulfur-oxidizing microorganisms (MAG 39 *Nitrobium versatile* and MAG 65 *Thiohalobacteraceae*), two key methane oxidizers (MAG 36 *Methanoperedens nitroreducens* and MAG 38 *Methylomirabilis tolerans*), and two key nitrogen-cycling microorganisms—the ammonium oxidizer *Ca. Kuenenia stuttgartiensis* (MAG 32) and a putative denitrifying *Acidobacterium* (MAG 60 *Thermoanaerobaculia 1*).

The *Nitrospirota* MAG 39 *Nitrobium versatile*, which was the most abundant microbial community member based on genome coverage before the ammonium removal experiment (Figure 3, G0), was estimated to be a 100% complete genome with 5% contamination (Figure 2). Based on metatranscriptomic analyses, we infer that the most likely metabolism performed by this organism was sulfide oxidation coupled to denitrification (Figure 4). A previously unidentified sulfide:quinone oxidoreductase *sqr* gene in *Ca. Nitrobium versatile* was one of the mostly highly transcribed functional genes (TPM = 0.2 ± 0.05) of this organism in a thriving period (T0, Supplementary Table 2), along with cytochrome *c*-oxidizing nitric oxide reductase genes *norBC* (respectively, TPM = 1.9 ± 0.7 and 2.8 ± 1.2). Sulfide could, thus, be oxidized to elemental sulfur, which might be the substrate for a dissimilatory sulfite reductase (*dsrABC*) operating in the oxidative direction, despite the two *dsrA* copies in the genome being phylogenetically related to reductive *dsrA* genes (Supplementary Figure 3) and the presence and transcription of a *dsrD* gene. Interestingly, both *dsrABC* copies in the genome were transcribed, one approximately three times more than the other (Supplementary Table 2, average TPM 0.3–0.4 vs. 0.1). Sulfite could be oxidized to sulfate *via* transcribed adenosine-phosphosulfate (APS) reductase (*aprAB*) and sulfate adenylyltransferase (*sat*) or *via* a sulfite:cytochrome *c* oxidoreductase (*sorAB*) (Figure 4). Although we did not provide reduced sulfur compounds to the reactor other than sulfide, two genes encoding putative sulfide-generating enzymes were identified and transcribed: a sulfhydrogenase polysulfide reductase (*hydB* subunit beta only, TPM = 0.1 ± 0.06), and a thiosulfate reductase/polysulfide reductase (chain A only, *phsA*, present twice in the genome, TPMs = 0.09 ± 0.05 and 0.016 ± 0.018). This organism was severely affected by the ammonium removal experiment, as indicated by genome coverage (Figure 3). TPM values suggest that *Ca. Nitrobium versatile* became a rare community member but remained transcriptionally active across all time points. When the reactor was primed for the sulfide and NO toxicity experiment (T4), transcripts for 161 genes were detected, including *norC* and *phsA* but not *sqr* or *norB*, while by the end of the experiment (T5), still, transcripts for 130 genes were found (Supplementary Table 2).

Interestingly, in MAG 39 *Nitrobium versatile*, several other functional genes were transcribed at low levels: membrane-bound and periplasmic nitrate reductase genes (*narGHI* and *napA*), ammonium-forming cytochrome *c*_552_-nitrite reductase (*nrfA*), and anaerobic dimethyl sulfoxide reductase (*dmsABC*). Although previously identified (Arshad et al., 2017), *napB* and *nirBD* were absent in the genome, and *nrfH* was present in the genome, but no transcription was detected. All genes encoding subunits of electron transport chain proteins were transcribed (Figure 4). Two genes encoding complexes with homology *caa*_3_-type low-affinity cytochrome *c* oxidase proteins in *Acidobacteria* were identified: (i) *ctaCFED*, which was followed by a downstream *sco* assembly protein-encoding gene and a cytochrome *c*_6_ (homologous to *petJ*, K08906), which we have putatively denominated *ctaX*, for it could be part of the complex, and (ii) *sco* followed by a downstream *ctaDEFC*. All these subunits were transcribed, except for *ctaF* of the latter complex. A Rieske *bc*_1_ complex was encoded by *petBCD*, which was preceded by a cytochrome *c* protein-encoding gene immediately upstream. Two other copies of *petBC* were present in the genome. All these subunits were transcribed except for the first *petB* (Supplementary Table 4). Finally, the MAG 39 *Nitrobium versatile* had two ammonium transporter-encoding genes (*amtB*-type), of which only the second was transcribed, solely in T0 (Supplementary Table 4).

The gammaproteobacterial MAG 65 *Thiohalobacteraceae*, representing an organism that seemed enriched over the sulfide and NO toxicity experiment (Figure 3), was estimated to be a 98.1% complete genome with 0.3% contamination (Figure 2). Based on metatranscriptomic analyses, we infer that the most likely metabolism performed by this organism was sulfide oxidation coupled to denitrification (Figure 4). A sulfide:quinone oxidoreductase-encoding *sqr* gene was among the functional genes with the highest transcription by the end of the sulfide and NO toxicity experiment (T5; TPM = 0.43 ± 0.36; Supplementary Tables 2,4). Also highly transcribed were the genes *dsrA* (TPM = 1.07 ± 0.78), *dsrB* (TPM = 0.95 ± 0.5), *dsrC* (TPM = 4.3 ± 1.1), as well as additional *dsrABC* copies. Sulfite oxidation to sulfate could proceed *via* proteins encoded by *sorA*, *soeABC* (quinone-sulfite dehydrogenase), as well as *aprAB* and *sat*, which were all transcribed. Sulfur oxidation system *soxBAZYX* genes for thiosulfate oxidation and a thiocyanate dehydrogenase *tcdh* gene were transcribed, indicating that these substrates could also be oxidized, although they were not provided to the reactor. No *soxCD* gene was identified in the genome. A cytochrome *cd*_1_-nitrite reductase *nirS* gene was highly transcribed (TPM = 1.74 ± 0.78), along with all other genes in the denitrification pathway, including a *nirK* gene encoding a copper-containing nitrite reductase. Ribulose-bisphosphate carboxylase *cbbSL* genes were among the most highly transcribed in the genome, and all genes encoding subunits of the electron transport chain were transcribed.

MAG 36 (*Ca. Methanoperedens nitroreducens*) was 99.4% complete with 4.6% contamination, and had all genes in the reverse methanogenesis pathway (Figure 4 and Supplementary Table 4) as well as potential for carbon fixation *via* the Wood–Ljungdahl pathway with carbon monoxide dehydrogenase/acetyl-CoA synthase (CODH/ACS)-encoding genes and acetate production or assimilation *via* an acetyl-CoA synthetase-encoding *acs* gene. All these genes were transcribed by the end of the sulfide and NO toxicity experiment (T5), as well as electron transport chain-encoding genes (Figure 4). The succinate dehydrogenase *sdhC* subunit was not present in the genome. Remarkably, the genome had genes encoding two copies of the membrane-bound nitrate reductase (*narGHI*), an ammonium-forming nitrite reductase (*nrfAH*), a non-electrogenic cytochrome *c*-oxidizing (cNOR) nitric oxide reductase (*norBC*), and an electrogenic quinone-oxidizing (qNOR) nitric oxide reductase (*norZ*). Methyl-coenzyme M reductase genes *mcrBDGA* had TPM values around ∼200–400 before the sulfide and NO toxicity experiment (T4), and ∼20–40 after (T5, Supplementary Table 2), indicating that, although *Ca. Methanoperedens nitroreducens* was still abundant by the end of the experiment (Figure 3), it suffered a degree of inhibition. Three hypothetical genes had highest transcription at T5 and were upregulated relative to T4 (Supplementary Table 2).

A second key methane oxidizer was represented by MAG 38 (*Ca*. *Methylomirabilis tolerans*), 94.5% complete with 2% contamination. This genome had all genes for carbon fixation *via* the Calvin cycle and for the methane oxidation *via* particulate methane monooxygenase (*pmoABC*) coupled to nitric oxide dismutation, with four putative *nod* genes present and transcribed. Additionally, nitrate, nitrite, and nitric oxide reductase-encoding genes were present and transcribed: *nxrABC*, ammonium-forming cytoplasmic NADH-nitrite reductase *nirBD* genes, electrogenic cytochrome *c*-oxidizing (sNOR) nitric oxide reductase-encoding *norSY* genes, non-electrogenic quinone-oxidizing (gNOR) nitric oxide reductase-encoding *norGH* genes, and *norZ*. All four genes encoding the two subunits of the putative nitric oxide dismutases NOD-1 and NOD-2 had some of the highest transcription (TPM ∼7–60) when the community was being primed for the sulfide and NO toxicity experiment, receiving ∼1–5% external NO in the reactor along with methane, ammonium, and sulfide (T4), as well as *pmoABC* (TPM ∼6–22) and the lanthanide-dependent methanol dehydrogenase-encoding *xoxF* gene (TPM = 5.5 ± 3.5) (Supplementary Table 2). Although *Ca*. *Methylomirabilis tolerans* was still abundant after the sulfide and NO toxicity experiment (Figure 3), TPM values for all aforementioned functional genes decreased 10- to 100-fold from T4 to T5, indicating that the microorganism suffered a degree of inhibition (Supplementary Table 2).

MAG 60 *Thermoanaerobaculia 1*, 88.3% complete with 1.5% contamination, had the highest normalized genome coverage when the reactor was being primed for the sulfide and NO toxicity experiment (Figure 3, G4). Based on metatranscriptomic analyses, the most likely metabolism performed by this organism was heterotrophic denitrification (Figure 4). A nitrous oxide reductase *nosZ* gene was among the functional genes with highest transcription (T4, TPM = 0.21 ± 0.17), followed by *narGHI* and *nrfAH*. Interestingly, hydrogenase-2 and low-affinity cytochrome *c* oxidase-encoding *ctaCDEF* genes were also transcribed, as well as all electron transport chain-encoding genes, including two RNF complexes (Supplementary Tables 2,4).

Finally, *Ca. Kuenenia stuttgartiensis*, represented by MAG 32, 95.6% complete with 0.6% contamination, was one of two microorganisms that persisted throughout all regular and experimental conditions (Figure 3). Interestingly, the transcription of hydrazine synthase *hzsABC* genes decreased 18–27× during the ammonium removal experiment (T1 to T2), and 2–5× during the sulfide and NO toxicity experiment (T4 to T5), suggesting that substrate deprivation was a stronger stressor than toxicity. MAG 32 had all genes encoding substrate oxidation and electron transport chain proteins in the anammox pathway (de Almeida et al., 2016), as well as carbon fixation *via* the Wood–Ljungdahl pathway, which were transcribed at all time points (Figure 4). The second microorganism that persisted throughout all regular and experimental conditions was represented by MAG 44, 93.1% complete with 3.4% contamination, a divergent *Planctomycetes* genome. Based on metagenomic and transcriptomic analyses, the most likely metabolism performed by this organism was heterotrophic denitrification *via narGHI* and *nirS* (Supplementary Table 2). While normalized genome coverage of this MAG varied only between 16× and 50× across regular and experimental conditions, summed TPM values for all genes transcribed in each time point were highest in the preparatory phase for the sulfide and NO toxicity experiment, as well as before and after the experiment (T3–5; TPM = ∼168–361) in comparison with previous time points (T0–2; TPM = ∼33–43; Supplementary Table 2).

### Microbial Community Transcriptional Responses to Substrate Removal and Toxicity Stresses Provide Insights Into Community Dynamics and Resiliency

Gene-centric analyses aimed to elucidate the transcriptional responses of the reactor community to two experimental conditions. The first, ammonium removal from the medium, while methane, sulfide, and nitrate were still provided to the reactor, tested the strength of microbial interactions for the supply of ammonium *via* DNRA to community members—in particular, to anammox bacteria. The second, sulfide and NO addition, while no other substrates were provided to the reactor, aimed to enrich sulfide-oxidizing nitric oxide reducers and to test the limits of microbial community resiliency to these stresses.

A major response to ammonium removal was a general inhibition of methane, ammonium, and sulfide oxidizers, as indicated by downregulation of their key genes *mcrA*, *pmoA*, *hzsA*, *sqr*, *soxB*, *sorA*, and *dsrABC* (Figure 5). RT-qPCR of *mcrA*, *pmoA*, and *hzsA* confirmed these trends (Supplementary Table 3). Not surprisingly, anammox bacteria were particularly affected, with an approximately 18-fold decrease in *hzsA* transcription. On the other hand, nitrogen fixation *nifDHK* genes, ammonium transporter *amtB* genes, and the ammonium-forming nitrite reductase *nrfA* genes were upregulated, likely to compensate for the ammonium limitation (Figure 5). *Ca. Methanoperedens nitroreducens* accounted for 97.6% of *nrfA* transcription and 48.2% of *amtB* transcription 10 weeks after ammonium was completely removed from the medium (T2). Other taxa that shared a large proportion of *amtB* transcription in T2 were *Ca. Methylomirabilis tolerans* (14.5%), *Ca. Methylomirabilis lanthanidiphila* (9.6%), and *Ca. Kuenenia stuttgartiensis* (3.2%). The *hzsA* gene of *Ca. Kuenenia stuttgartiensis* had at least 20× higher transcription than *hzsA* of *Ca. Scalindua rubra* across all time points, although *Ca. Scalindua rubra* had higher genome coverage before experiments were conducted (Figure 3). Finally, minor changes included the upregulation of the periplasmic nitrate reductase *napA*, *nirB*, *soeA*, and quinone-thiosulfate dehydrogenase *doxD* genes.

A major response to the sulfide and NO toxicity experiment was upregulation of all sulfur oxidation genes (*sqr*, *soxB*, *sorA*, soeA, and *dsrABC*) (Figure 5). MAG 65 *Thiohalobacteraceae* accounted for 67% of *sqr* transcription by the end of the experiment (T5). Interestingly, the transcription of *norB* genes decreased 32 times. However, *norB* TPM values were the highest among all nitrogen cycle marker genes across all time points in this study except T5 in which *hzsA* (average TPM = 7) and *norB* (average TPM = 5.22) TPM values were similarly low. *Ca. Methylomirabilis tolerans* accounted for 43% of *norB* transcription at T5, which included NOD-1 (18%), NOD-2 (19%), and qNOR (6%)-encoding genes, followed by *Ca. Methanoperedens nitroreducens* (21%, with the qNOR-encoding gene accounting for 13% and cNOR-encoding gene for 8%), *Ca. Methylomirabilis lanthanidiphila* (10%), and MAG 65 *Thiohalobacteraceae* (9.6%). The transcription of several other genes encoding proteins involved in denitrification (*narG*, *napA*, *nirS*, and *nirB*) was downregulated. However, *nirK* had a 12-fold increase in transcription, and *nosZ* had a minor increase. Methane oxidation marker genes were downregulated, as indicated by a 19-fold decrease in *mcrA* transcription and 21-fold decrease in *pmoA* transcription. Transcription of the anammox marker gene *hzsA* decreased 2.7-fold. RT-qPCR of *mcrA*, *pmoA*, and *hzsA* confirmed these trends (Supplementary Table 3). Carbon fixation *via* CODH/ACS was downregulated, but CO_2_ fixation *via* ribulose–bisphosphate carboxylase–oxygenase was upregulated.

## Discussion

In this study, we conducted metagenomic and metatranscriptomic analyses in order to unravel metabolic pathways, microbial dynamics, and responses to stresses in a bioreactor community mimicking anoxic and brackish coastal sediment conditions. Microbial interactions and resiliency were tested by removal and addition of key substrates. Removal of ammonium from the medium had major effects on microbial activity and community structure: *Ca. Methanoperedens nitroreducens* dominated DNRA activity (Figure 5), and after ammonium was reintroduced in the medium, became the most abundant community member (Figure 3). *Ca. Kuenenia stuttgartiensis* replaced *Ca. Scalindua rubra* as the most abundant anammox bacterium, several sulfide oxidizers replaced *Ca. Nitrobium versatile*, and *Ca. Methylomirabilis tolerans* dominated over *Ca. Methylomirabilis lanthanidiphila* (Figures 3, 5). These results suggest strong microbial cooperation between *Ca. Methanoperedens nitroreducens* and *Ca. Methylomirabilis* species for the exchange of nitrite under the abundance of methane. Before the ammonium removal experiment, TPM values indicate that *Ca. Methanoperedens nitroreducens* reduced nitrate to nitrite, which was reduced to nitric oxide mostly by *Ca. Methylomirabilis lanthanidiphila*. Nitric oxide was then shared among three main organisms: *Ca. Methylomirabilis lanthanidiphila*, *Ca. Methylomirabilis tolerans*, and *Ca. Nitrobium versatile*. However, under ammonium limitation, *Ca. Methanoperedens nitroreducens* further reduced nitrite to ammonium *via* DNRA, and this had a cascade effect that changed microbial community structure, resulting in *Ca. Methylomirabilis tolerans* dominating and *Ca. Methylomirabilis lanthanidiphila* and *Ca. Nitrobium versatile* concomitantly decreasing in abundance and activity. These results suggest that *Ca. Methylomirabilis tolerans* was a more competitive organism in scavenging nitric oxide. However, the mechanisms remain to be elucidated. Both *Ca. Methylomirabilis* species encoded only a lanthanide-dependent methanol dehydrogenase and could be enriched in the bioreactor due to cerium being provided as part of the medium (Versantvoort et al., 2018, 2019; Guerrero-Cruz et al., 2019).

Anammox bacteria were more strongly limited by ammonium removal than *Ca. Methylomirabilis* species, presumably due to the dual competition for both ammonium and nitrite, the key substrates for anammox energy metabolism. Although *Ca. Methylomirabilis* organisms have a lower affinity for nitrite [K_s_ = 7 μM; *Ca. M. oxyfera* and *Ca. M. lanthanidiphila* (Guerrero-Cruz et al., 2019)] than anammox bacteria [K_s_ = 0.2–3 μM; *Ca. K. stuttgartiensis* and *Ca. Scalindua sp*. (Oshiki et al., 2016)], additional competition for ammonium could have caused anammox to be less fit. Yet, *Ca. K. stuttgartiensis* [K_s_ < 5 μM for ammonium (Strous et al., 1999)] seemed to better withstand ammonium starvation than *Ca. Scalindua rubra* [K_s_ = 3 μM for ammonium in several *Ca. Scalindua* species (Oshiki et al., 2016)], indicating that the affinity of *Ca*. *K. stuttgartiensis* for ammonium could be higher than that of *Ca*. *Scalindua*. *Ca. Nitrobium versatile*, on the other hand, seemed to rely on nitric oxide for sulfide oxidation, decreasing in abundance as *Ca. Methanoperedens nitroreducens* [Ks = 2.1 ± 0.4 mg N L^–1^ for nitrate (Lu et al., 2019)], and *Ca. Methylomirabilis tolerans* increased (Figure 3). All three organisms had potential to generate ammonium (Figure 4), but *Ca. Methanoperedens nitroreducens* dominated DNRA activity (Figure 5). Therefore, we conclude that DNRA from *Ca. M. nitroreducens* alone could not sustain anammox activity under ammonium, nitrite, and organic carbon (except methane) limitation, favoring *Ca. Methylomirabilis tolerans*, which likely outcompeted *Ca. N. versatile* and anammox bacteria for nitrite and nitric oxide. These results are in contrast to estuary ecosystems in which anammox activity could be sustained by DNRA, positively correlating to sulfide and sediment organic carbon content (Lisa et al., 2014). High sediment organic carbon to nitrate ratio and ferrous iron availability have been reported to favor DNRA over denitrification in estuary ecosystems (Kessler et al., 2018). Having methane as the only organic carbon source (at saturation) and a high nitrate load (3 mmol/day) could have, thus, modulated DNRA activity to become insufficient to sustain anammox and changed microbial community structure.

We also targeted the enrichment of sulfide-oxidizing NO reducers while characterizing microbial community responses to sulfide and NO toxicity. We found genomic potential and transcriptional evidence for this metabolism in *Ca. Nitrobium versatile*, the dominant microorganism in the bioreactor community before the ammonium removal experiment (Figure 3). However, *Ca. Nitrobium versatile* became a rare community member after this experiment and could no longer be enriched. Instead, MAG 65 *Thiohalobacteraceae*, representing an organism likely performing sulfide oxidation coupled to denitrification, increased in abundance (Figures 3–5). The sulfide and NO toxicity experiment revealed unusual resiliency of *Ca. M. nitroreducens* and *Ca. M. tolerans*, which persisted as the most abundant community members (Figure 3) even though methane and nitrate were no longer provided to the reactor for the 7 weeks of the experiment, consistent with the downregulation of *mcrA* and *pmoA* (Figure 5 and Supplementary Table 3). Given that the hydraulic retention time of the reactor was 5 days, it is unlikely that our sequencing results and the dominance of *Ca. M. nitroreducens* and *Ca. M. tolerans* simply reflect older decaying biomass, which would have been removed.

MAG 65 *Thiohalobacteraceae*, MAG 44 *Planctomycetes 1*, MAG 60 *Thermoanaerobaculia 1*, and *Ca*. *Kuenenia stuttgartiensis* followed as the most abundant community members at the end of the experiment. While the first three could make use of sulfide or organic carbon from decaying biomass as electron donors and residual nitrate or nitrite and nitric oxide as electron acceptors, *Ca. K. stuttgartiensis* could have used ammonium generated from decaying biomass and nitrite from residual nitrate reduction or nitric oxide (Hu et al., 2019). Of these four MAGs, only MAG 65 *Thiohalobacteraceae* increased in abundance and had both *nirK* and *norB* transcription increased during the sulfide and NO toxicity experiment (Figure 5 and Supplementary Table 2), while the other three suffered a degree of inhibition inferred from both decreases in genome coverage (Figure 3) and marker gene transcriptional activity (Figure 5 and Supplementary Table 2). Given that sulfide remained below detection limit (0.15 μM) during all time points, we infer that this inhibition is attributed to nitrite (∼100–400 μM) and NO (∼10–13% of headspace). While nitrite and NO were likely toxic for several community members, MAG 65 *Thiohalobacteraceae* seemed to have taken advantage of these compounds as terminal electron acceptors.

MAG 65 *Thiohalobacteraceae* had similar metabolic potential as the gammaproteobacterium *Thiohalobacter thiocyanaticus*, which has sulfur oxidation genes encoding FccAB, DsrABC, AprAB, Sat, SoeABC, and SoxXABYZ, as well as thiocyanate dehydrogenase and carbon fixation *via* the Calvin cycle (Tsallagov et al., 2019). Additionally, MAG 65 had three *sqr* copies and two *sorA* copies. Interestingly, MAG 39 *N. versatile* also had a sulfur oxidation pathway that included *sqr*, *dsrABC*, *sorA* or *aprAB*, and *sat*. Umezawa et al. (2020) described the YTD gene cluster composed of genes *yedE*-like, *tusA*, *dsrE*-like, *chp-1*, and *chp-2*, which encoded proteins for sulfur disproportionation in *Nitrospirota* (Umezawa et al., 2020). As in this previous study, we also detected an incomplete YTD gene cluster in *Ca. N. versatile* (MAG 39), indicating that it might lack sulfur disproportionation potential *via* this cluster, in contrast to the *Nitrospirota* microorganism species 45J (Umezawa et al., 2020), which shares 64% average amino acid identity (AAI) to *Ca. N. versatile*. The lack of a complete YTD gene cluster is also described in the *Nitrospirota* microorganism *Ca. Sulfobium mesophilum* (Zecchin et al., 2018; Umezawa et al., 2020), which has *sqr* and *dsrABCD* as well as *napAB* and *nrfAH*, and shares 57% average amino acid identity to MAG 39 *N. versatile*. These AAI values indicate that *Ca. N. versatile* and species 45J are likely part of the same *Ca. Nitrobium* genus but distinct species, and that together with *Ca. Sulfobium mesophilum*, they form a family-level taxonomic group (Luo et al., 2014). While conducting our study, Umezawa et al. (2021) isolated a sulfur-disproportionating microorganism that was named *Dissulfurispira thermophila*, matching the previously described genus *Ca. Nitrobium* (Arshad et al., 2017).

*Ca. Sulfobium mesophilum* and *Ca. N. versatile* both have *dsrA* genes that affiliate with bacterial-type reductive sequences, and a *dsrD* gene, suggested as a potential marker for sulfate reduction, absent in sulfur oxidizers that utilize the reverse Dsr pathway (Rabus et al., 2015; Anantharaman et al., 2018; Dalcin Martins et al., 2018). However, our metatranscriptomic results suggest that *Ca. N. versatile* was performing sulfide oxidation. Therefore, it seems that *Ca. N. versatile*, similar to the deltaproteobacterium *Desulfurivibrio alkaliphilus*, was yet another example of a microorganism disguised as a sulfate reducer (Thorup et al., 2017). Interestingly, *Ca. Sulfobium mesophilum* and *Desulfurivibrio alkaliphilus* could couple sulfide oxidation to DNRA, a metabolic potential also present in MAG 39, with transcripts of *nrfA* detected in our study (T0; TPM = 0.030 ± 0.031). Given that *norBC* had significantly higher transcriptional activity than *nrfA* and other denitrification genes (T0, TPM *norB* = 1.9 ± 0.7, TPM *norC* = 2.8 ± 1.1), it is more likely that *Ca. N. versatile* coupled sulfide oxidation to nitric oxide reduction. Given that *Ca. N. versatile* could not be enriched under sulfide and NO, we hypothesize that MAG 65 *Thiohalobacteraceae* represented a more competitive microorganism. However, the mechanism and substrate affinities remain to be elucidated.

In our study, methanotrophs could withstand prolonged periods of ammonium, methane, and nitrate deprivation as well as exposure to elevated concentrations of nitrite and nitric oxide. During these disturbances, *Ca. Methanoperedens nitroreducens* and *Ca. Methylomirabilis tolerans* did not thrive, as indicated by downregulation of *mcrA* and *pmoA*, but tolerated stresses and persisted as abundant community members. These results suggest that methane oxidation could be a relatively stable community function in coastal ecosystems under the stresses investigated in this study and as long as the system stays anoxic. Future investigations are needed to elucidate such dynamics. Interestingly, the *Ca. Methanoperedens nitroreducens* genome (MAG 36) encoded nitric oxide reductases (Figure 4), which had not been described before in this organism. The qNOR-encoding gene, present in the same contig as *mcr*, *nar*, and *nrf* genes, had increased transcriptional activity by the end of the sulfide and NO stress experiment (Figure 5). Future studies should further evaluate lateral transfer of *nor* genes in *Methanoperedenaceae*, which seem prone to acquire novel metabolic traits *via* later gene transfer events (Leu et al., 2020), potentially *via* recently described borgs (Al-Shayeb et al., 2021), as well as metabolic flexibility of *Ca. Methanoperedens nitroreducens* under methane deprivation. Hypothetical genes in *Ca. Methanoperedens nitroreducens* identified in this study with high transcription and upregulation in response to stresses could be targets for these future investigations.

Sulfide oxidizers had differential resilience to the stresses investigated in this study, dynamically changing in abundance and transcriptional activity across regular and experimental conditions. However, sulfide was completely removed at all time points, likely due to functional redundancy in the microbial community, indicating that sulfide oxidation could also be a relatively stable community function under the investigated stresses. Finally, in our study, denitrification was a dominant nitrogen-cycling pathway, as previously suggested in coastal sediments of the Bothnian Sea (Rasigraf et al., 2019)—particularly, the nitric oxide reduction step, as indicated by *norB* TPM values (Supplementary Table 2). Anammox activity, as indicated by *hzsA* TPM values, also had a significant contribution to nitrogen cycling, and dominated at the end of the sulfide and nitric oxide stress experiment (T5). This community function was highly impacted by ammonium deprivation, but was restored when favorable conditions were reestablished. On the other hand, DNRA, as indicated by *nrfA* TPM values, was a minor nitrogen-cycling pathway, but under ammonium deprivation, it became more significant. Future studies should investigate whether anammox and DNRA similarly oscillate in coastal ecosystems under the stresses investigated in this study.

## Conclusion

This study contributes to the elucidation of metabolic pathways for carbon, sulfur, and nitrogen cycling in a bioreactor community mimicking anoxic and brackish coastal sediment conditions, as well as shifts in microbial abundance and transcriptional activity in response to prolonged substrate deprivation and exposure to toxic compounds. Together with follow-up studies, these results will help in understanding complex microbial interactions and functions in dynamic coastal ecosystems, and should be considered into future modeling efforts that aim to predict coastal ecosystem responses to environmental change.

## Data Availability Statement

The datasets presented in this study can be found in online repositories. The names of the repository/repositories and accession number(s) can be found in the article/Supplementary Material.

## Author Contributions

HJMO, MSMJ, CUW, and PDM planned and designed the study. PDM, MJEM, JMK, AA, and HTO executed the research project. PDM and MJEM wrote the manuscript with input from all co-authors. All authors read and approved the final version submitted.

## Conflict of Interest

The authors declare that the research was conducted in the absence of any commercial or financial relationships that could be construed as a potential conflict of interest.

## Publisher’s Note

All claims expressed in this article are solely those of the authors and do not necessarily represent those of their affiliated organizations, or those of the publisher, the editors and the reviewers. Any product that may be evaluated in this article, or claim that may be made by its manufacturer, is not guaranteed or endorsed by the publisher.

## Footnotes

1
http://enve-omics.ce.gatech.edu/g-matrix/index

2
https://ggdc.dsmz.de/ggdc.php#

## References

[B1] Alneberg J. Bjarnason B. S. de Bruijn I. Schirmer M. Quick J. Ijaz U. Z. (2014). Binning metagenomic contigs by coverage and composition. *Nat. Methods* 11 1144–1146. 10.1038/nmeth.3103 25218180

[B2] Al-Shayeb B. Schoelmerich M. C. West-Roberts J. Valentin-Alvarado L. E. Sachdeva R. Mullen S. (2021). Borgs are giant extrachromosomal elements with the potential to augment methane oxidation. *bioRxiv* [preprint]. 10.1101/2021.07.10.451761

[B3] Anantharaman K. Hausmann B. Jungbluth S. P. Kantor R. S. Lavy A. Warren L. A. (2018). Expanded diversity of microbial groups that shape the dissimilatory sulfur cycle. *ISME J.* 12 1715–1728. 10.1038/s41396-018-0078-0 29467397 PMC6018805

[B4] Arshad A. Dalcin Martins P. Frank J. Jetten M. S. M. Op den Camp H. J. M. Welte C. U. (2017). Mimicking microbial interactions under nitrate-reducing conditions in an anoxic bioreactor: enrichment of novel Nitrospirae bacteria distantly related to *Thermodesulfovibrio*. *Environ. Microbiol.* 19 4965–4977. 10.1111/1462-2920.13977 29105249

[B5] Bürgmann H. Jenni S. Vazquez F. Udert K. M. (2011). Regime shift and microbial dynamics in a sequencing batch reactor for nitrification and Anammox treatment of urine. *Appl. Environ. Microbiol.* 77 5897–5907. 10.1128/AEM.02986-10 21724875 PMC3165423

[B6] Bushnell B. (2016). *BBMap Short Read Aligner.* Available online at: https://sourceforge.net/projects/bbmap/ (accessed February 9, 2021).

[B7] Caffrey J. M. Bonaglia S. Conley D. J. (2019). Short exposure to oxygen and sulfide alter nitrification, denitrification, and DNRA activity in seasonally hypoxic estuarine sediments. *FEMS Microbiol. Lett.* 366:fny288. 10.1093/femsle/fny288 30596977 PMC6343015

[B8] Capella-Gutierrez S. Silla-Martinez J. M. Gabaldon T. (2009). trimAl: a tool for automated alignment trimming in large-scale phylogenetic analyses. *Bioinformatics* 25 1972–1973. 10.1093/bioinformatics/btp348 19505945 PMC2712344

[B9] Chaumeil P.-A. Mussig A. J. Hugenholtz P. Parks D. H. (2019). GTDB-Tk: a toolkit to classify genomes with the Genome Taxonomy Database. *Bioinformatics* 36 1925–1927. 10.1093/bioinformatics/btz848 31730192 PMC7703759

[B10] Chen J. Hanke A. Tegetmeyer H. E. Kattelmann I. Sharma R. Hamann E. (2017). Impacts of chemical gradients on microbial community structure. *ISME J.* 11 920–931. 10.1038/ismej.2016.175 28094795 PMC5363838

[B11] Dalcin Martins P. Danczak R. E. Roux S. Frank J. Borton M. A. Wolfe R. A. (2018). Viral and metabolic controls on high rates of microbial sulfur and carbon cycling in wetland ecosystems. *Microbiome* 6:138. 10.1186/s40168-018-0522-4 30086797 PMC6081815

[B12] de Almeida N. M. Wessels H. J. C. T. de Graaf R. M. Ferousi C. Jetten M. S. M. Keltjens J. T. (2016). Membrane-bound electron transport systems of an anammox bacterium: a complexome analysis. *Biochim. Biophys. Acta Bioenerg.* 1857 1694–1704. 10.1016/j.bbabio.2016.07.006 27461995

[B13] Delgado Vela J. Bristow L. A. Marchant H. K. Love N. G. Dick G. J. (2021). Sulfide alters microbial functional potential in a methane and nitrogen cycling biofilm reactor. *Environ. Microbiol.* 23 1481–1495. 10.1111/1462-2920.15352 33295079

[B14] Deng Y.-F. Wu D. Huang H. Cui Y.-X. van Loosdrecht M. C. M. Chen G.-H. (2021). Exploration and verification of the feasibility of sulfide-driven partial denitrification coupled with anammox for wastewater treatment. *Water Res.* 193:116905. 10.1016/j.watres.2021.116905 33581404

[B15] Edgar R. C. (2004). MUSCLE: multiple sequence alignment with high accuracy and high throughput. *Nucleic Acids Res.* 32 1792–1797. 10.1093/nar/gkh340 15034147 PMC390337

[B16] Egger M. Riedinger N. Mogollón J. M. Jørgensen B. B. (2018). Global diffusive fluxes of methane in marine sediments. *Nat. Geosci.* 11 421–425. 10.1038/s41561-018-0122-8

[B17] Engelbrektson A. Kunin V. Wrighton K. C. Zvenigorodsky N. Chen F. Ochman H. (2010). Experimental factors affecting PCR-based estimates of microbial species richness and evenness. *ISME J.* 4 642–647. 10.1038/ismej.2009.153 20090784

[B18] Ettwig K. F. Zhu B. Speth D. Keltjens J. T. Jetten M. S. M. Kartal B. (2016). Archaea catalyze iron-dependent anaerobic oxidation of methane. *Proc. Natl. Acad. Sci. U.S.A.* 113 12792–12796. 10.1073/pnas.1609534113 27791118 PMC5111651

[B19] Graf J. S. Mayr M. J. Marchant H. K. Tienken D. Hach P. F. Brand A. (2018). Bloom of a denitrifying methanotroph, ‘ *Candidatus Methylomirabilis limnetica’*, in a deep stratified lake. *Environ. Microbiol.* 20 2598–2614. 10.1111/1462-2920.14285 29806730

[B20] Grieshaber M. K. Völkel S. (1998). Animal adaptations for tolerance and exploitation of poisonous sulfide. *Annu. Rev. Physiol.* 60 33–53. 10.1146/annurev.physiol.60.1.33 9558453

[B21] Guerrero-Cruz S. Stultiens K. van Kessel M. A. H. J. Versantvoort W. Jetten M. S. M. Op den Camp H. J. M. (2019). Key physiology of a nitrite-dependent methane-oxidizing enrichment culture. *Appl. Environ. Microbiol.* 85:e00124-19. 10.1128/AEM.00124-19 30770408 PMC6450021

[B22] Harhangi H. R. Le Roy M. van Alen T. Hu B. Groen J. Kartal B. (2012). Hydrazine synthase, a unique phylomarker with which to study the presence and biodiversity of anammox bacteria. *Appl. Environ. Microbiol.* 78 752–758. 10.1128/AEM.07113-11 22138989 PMC3264106

[B23] Howarth R. W. Marino R. (2006). Nitrogen as the limiting nutrient for eutrophication in coastal marine ecosystems: evolving views over three decades. *Limnol. Oceanogr.* 51 364–376. 10.4319/lo.2006.51.1_part_2.0364

[B24] Hu Z. Wessels H. J. C. T. van Alen T. Jetten M. S. M. Kartal B. (2019). Nitric oxide-dependent anaerobic ammonium oxidation. *Nat. Commun.* 10:1244. 10.1038/s41467-019-09268-w 30886150 PMC6423088

[B25] Hyatt D. Chen G.-L. LoCascio P. F. Land M. L. Larimer F. W. Hauser L. J. (2010). Prodigal: prokaryotic gene recognition and translation initiation site identification. *BMC Bioinformatics* 11:119. 10.1186/1471-2105-11-119 20211023 PMC2848648

[B26] Joshi N. A. Fass J. N. (2011). *Sickle: A Sliding-Window, Adaptive, Quality-Based Trimming Tool for FastQ Files.* Available online at: https://github.com/najoshi/sickle (accessed February 9, 2021).

[B27] Kang D. D. Li F. Kirton E. Thomas A. Egan R. An H. (2019). MetaBAT 2: an adaptive binning algorithm for robust and efficient genome reconstruction from metagenome assemblies. *PeerJ* 7:e7359. 10.7717/peerj.7359 31388474 PMC6662567

[B28] Kessler A. J. Roberts K. L. Bissett A. Cook P. L. M. (2018). Biogeochemical controls on the relative importance of denitrification and dissimilatory nitrate reduction to ammonium in estuaries. *Glob. Biogeochem. Cycles* 32 1045–1057. 10.1029/2018GB005908

[B29] Langmead B. Salzberg S. L. (2012). Fast gapped-read alignment with Bowtie 2. *Nat. Methods* 9 357–359. 10.1038/nmeth.1923 22388286 PMC3322381

[B30] Letunic I. Bork P. (2021). Interactive Tree Of Life (iTOL) v5: an online tool for phylogenetic tree display and annotation. *Nucleic Acids Res.* 49 W293–W296. 10.1093/nar/gkab301 33885785 PMC8265157

[B31] Leu A. O. McIlroy S. J. Ye J. Parks D. H. Orphan V. J. Tyson G. W. (2020). Lateral gene transfer drives metabolic flexibility in the anaerobic methane-oxidizing archaeal family methanoperedenaceae. *MBio* 11:e01325-20. 10.1128/mBio.01325-20 32605988 PMC7327174

[B32] Li D. Luo R. Liu C.-M. Leung C.-M. Ting H.-F. Sadakane K. (2016). MEGAHIT v1.0: a fast and scalable metagenome assembler driven by advanced methodologies and community practices. *Methods* 102 3–11. 10.1016/j.ymeth.2016.02.020 27012178

[B33] Lisa J. Song B. Tobias C. Duernberger K. (2014). Impacts of freshwater flushing on anammox community structure and activities in the New River Estuary, USA. *Aquat. Microb. Ecol.* 72 17–31. 10.3354/ame01682

[B34] Lu P. Liu T. Ni B.-J. Guo J. Yuan Z. Hu S. (2019). Growth kinetics of *Candidatus* ‘*Methanoperedens nitroreducens’* enriched in a laboratory reactor. *Sci. Total Environ.* 659 442–450. 10.1016/j.scitotenv.2018.12.351 31096374

[B35] Luo C. Rodriguez-R L. M. Konstantinidis K. T. (2014). MyTaxa: an advanced taxonomic classifier for genomic and metagenomic sequences. *Nucleic Acids Res.* 42:e73. 10.1093/nar/gku169 24589583 PMC4005636

[B36] Madsen E. L. (2011). Microorganisms and their roles in fundamental biogeochemical cycles. *Curr. Opin. Biotechnol.* 22 456–464. 10.1016/j.copbio.2011.01.008 21333523

[B37] Malone T. C. Newton A. (2020). The globalization of cultural eutrophication in the coastal ocean: causes and consequences. *Front. Mar. Sci.* 7:670. 10.3389/fmars.2020.00670

[B38] Meier-Kolthoff J. P. Auch A. F. Klenk H.-P. Göker M. (2013). Genome sequence-based species delimitation with confidence intervals and improved distance functions. *BMC Bioinformatics* 14:60. 10.1186/1471-2105-14-60 23432962 PMC3665452

[B39] Moest R. R. (1975). Hydrogen sulfide determination by the methylene blue method. *Anal. Chem.* 47 1204–1205. 10.1021/ac60357a008

[B40] Na S.-I. Kim Y. O. Yoon S.-H. Ha S. Baek I. Chun J. (2018). UBCG: up-to-date bacterial core gene set and pipeline for phylogenomic tree reconstruction. *J. Microbiol.* 56 280–285. 10.1007/s12275-018-8014-6 29492869

[B41] Nazaries L. Pan Y. Bodrossy L. Baggs E. M. Millard P. Murrell J. C. (2013). Evidence of microbial regulation of biogeochemical cycles from a study on methane flux and land use change. *Appl. Environ. Microbiol.* 79 4031–4040. 10.1128/AEM.00095-13 23624469 PMC3697577

[B42] Nie W.-B. Ding J. Xie G.-J. Tan X. Lu Y. Peng L. (2021). Simultaneous nitrate and sulfate dependent anaerobic oxidation of methane linking carbon, nitrogen and sulfur cycles. *Water Res.* 194:116928. 10.1016/j.watres.2021.116928 33618110

[B43] Oshiki M. Satoh H. Okabe S. (2016). Ecology and physiology of anaerobic ammonium oxidizing bacteria. *Environ. Microbiol.* 18 2784–2796. 10.1111/1462-2920.13134 26616750

[B44] Parks D. H. Imelfort M. Skennerton C. T. Hugenholtz P. Tyson G. W. (2015). CheckM: assessing the quality of microbial genomes recovered from isolates, single cells, and metagenomes. *Genome Res.* 25 1043–1055. 10.1101/gr.186072.114 25977477 PMC4484387

[B45] Price M. N. Dehal P. S. Arkin A. P. (2010). FastTree 2 – Approximately maximum-likelihood trees for large alignments. *PLoS One* 5:e9490. 10.1371/journal.pone.0009490 20224823 PMC2835736

[B46] Rabus R. Venceslau S. S. Wöhlbrand L. Voordouw G. Wall J. D. Pereira I. A. C. (2015). A post-genomic view of the ecophysiology, catabolism and biotechnological relevance of sulphate-reducing prokaryotes. *Adv. Microb. Physiol.* 66 55–321. 10.1016/bs.ampbs.2015.05.002 26210106

[B47] Rasigraf O. Helmond N. A. G. M. Frank J. Lenstra W. K. Egger M. Slomp C. P. (2019). Microbial community composition and functional potential in Bothnian Sea sediments is linked to Fe and S dynamics and the quality of organic matter. *Limnol. Oceanogr.* 65 S113–S133. 10.1002/lno.11371

[B48] Rodriguez-R L. M. Konstantinidis K. T. (2016). The enveomics collection: a toolbox for specialized analyses of microbial genomes and metagenomes. *PeerJ Preprints* 4:e1900v1. 10.7287/peerj.preprints.1900v1

[B49] Russ L. Speth D. R. Jetten M. S. M. Op den Camp H. J. M. Kartal B. (2014). Interactions between anaerobic ammonium and sulfur-oxidizing bacteria in a laboratory scale model system. *Environ. Microbiol.* 16 3487–3498. 10.1111/1462-2920.12487 24750895

[B50] Saad S. Bhatnagar S. Tegetmeyer H. E. Geelhoed J. S. Strous M. Ruff S. E. (2017). Transient exposure to oxygen or nitrate reveals ecophysiology of fermentative and sulfate-reducing benthic microbial populations. *Environ. Microbiol.* 19 4866–4881. 10.1111/1462-2920.13895 28836729 PMC5763382

[B51] Shade A. Peter H. Allison S. D. Baho D. L. Berga M. Bürgmann H. (2012). Fundamentals of microbial community resistance and resilience. *Front. Microbiol.* 3:417. 10.3389/fmicb.2012.00417 23267351 PMC3525951

[B52] Shaffer M. Borton M. A. McGivern B. B. Zayed A. A. La Rosa S. L. Solden L. M. (2020). DRAM for distilling microbial metabolism to automate the curation of microbiome function. *Nucleic Acids Res.* 48 8883–8900. 10.1093/nar/gkaa621 32766782 PMC7498326

[B53] Sieber C. M. K. Probst A. J. Sharrar A. Thomas B. C. Hess M. Tringe S. G. (2018). Recovery of genomes from metagenomes *via* a dereplication, aggregation and scoring strategy. *Nat. Microbiol.* 3 836–843. 10.1038/s41564-018-0171-1 29807988 PMC6786971

[B54] Strous M. Kuenen J. G. Jetten M. S. (1999). Key physiology of anaerobic ammonium oxidation. *Appl. Environ. Microbiol.* 65 3248–3250. 10.1128/AEM.65.7.3248-3250.1999 10388731 PMC91484

[B55] Taylor S. Ninjoor V. Dowd D. M. Tappel A. L. (1974). Cathepsin B2 measurement by sensitive fluorometric ammonia analysis. *Anal. Biochem.* 60 153–162. 10.1016/0003-2697(74)90140-7 4850914

[B56] Thorup C. Schramm A. Findlay A. J. Finster K. W. Schreiber L. (2017). Disguised as a sulfate reducer: growth of the deltaproteobacterium *Desulfurivibrio alkaliphilus* by Sulfide Oxidation with Nitrate. *MBio* 8:e00671-17. 10.1128/mBio.00671-17 28720728 PMC5516251

[B57] Treseder K. K. Balser T. C. Bradford M. A. Brodie E. L. Dubinsky E. A. Eviner V. T. (2012). Integrating microbial ecology into ecosystem models: challenges and priorities. *Biogeochemistry* 109 7–18. 10.1007/s10533-011-9636-5

[B58] Tsallagov S. I. Sorokin D. Y. Tikhonova T. V. Popov V. O. Muyzer G. (2019). Comparative genomics of *Thiohalobacter thiocyanaticus* HRh1T and *Guyparkeria* sp. SCN-R1, halophilic chemolithoautotrophic sulfur-oxidizing gammaproteobacteria capable of using thiocyanate as energy source. *Front. Microbiol.* 10:898. 10.3389/fmicb.2019.00898 31118923 PMC6504805

[B59] Umezawa K. Kojima H. Kato Y. Fukui M. (2020). Disproportionation of inorganic sulfur compounds by a novel autotrophic bacterium belonging to *Nitrospirota*. *Syst. Appl. Microbiol.* 43:126110. 10.1016/j.syapm.2020.126110 32847785

[B60] Umezawa K. Kojima H. Kato Y. Fukui M. (2021). *Dissulfurispira thermophila* gen. nov., sp. nov., a thermophilic chemolithoautotroph growing by sulfur disproportionation, and proposal of novel taxa in the phylum *Nitrospirota* to reclassify the genus *Thermodesulfovibrio*. *Syst. Appl. Microbiol.* 44:126184. 10.1016/j.syapm.2021.126184 33676265

[B61] Vaksmaa A. Jetten M. S. M. Ettwig K. F. Lüke C. (2017). McrA primers for the detection and quantification of the anaerobic archaeal methanotroph ‘*Candidatus Methanoperedens nitroreducens*’. *Appl. Microbiol. Biotechnol.* 101 1631–1641. 10.1007/s00253-016-8065-8 28084539 PMC5266762

[B62] Van Helmond N. A. G. M. Robertson E. K. Conley D. J. Humborg C. Kubeneck L. J. Lenstra W. K. (2020). Removal of phosphorus and nitrogen in sediments of the eutrophic Stockholm Archipelago, Baltic Sea. *Biogeosciences* 17, 2745–2766. 10.5194/bg-17-2745-2020

[B63] Versantvoort W. Guerrero-Castillo S. Wessels H. J. C. T. van Niftrik L. Jetten M. S. M. Brandt U. (2019). Complexome analysis of the nitrite-dependent methanotroph *Methylomirabilis lanthanidiphila*. *Biochim. Biophys. Acta Bioenerg.* 1860 734–744. 10.1016/j.bbabio.2019.07.011 31376363

[B64] Versantvoort W. Guerrero-Cruz S. Speth D. R. Frank J. Gambelli L. Cremers G. (2018). Comparative genomics of *Candidatus methylomirabilis* species and description of Ca. *Methylomirabilis lanthanidiphila*. *Front. Microbiol.* 9:1672. 10.3389/fmicb.2018.01672 30140258 PMC6094997

[B65] Wallenius A. J. Dalcin Martins P. Slomp C. P. Jetten M. S. M. (2021). Anthropogenic and environmental constraints on the microbial methane cycle in coastal sediments. *Front. Microbiol.* 12:631621. 10.3389/fmicb.2021.631621 33679659 PMC7935538

[B66] Wells N. S. Chen J.-J. Maher D. T. Huang P. Erler D. V. Hipsey M. (2020). Changing sediment and surface water processes increase CH4 emissions from human-impacted estuaries. *Geochim. Cosmochim. Acta* 280 130–147. 10.1016/j.gca.2020.04.020

[B67] Wu Y. W. Simmons B. A. Singer S. W. (2015). MaxBin 2.0: an automated binning algorithm to recover genomes from multiple metagenomic datasets. *Bioinformatics* 32 605–607. 10.1093/bioinformatics/btv638 26515820

[B68] Zecchin S. Mueller R. C. Seifert J. Stingl U. Anantharaman K. von Bergen M. (2018). Rice paddy nitrospirae carry and express genes related to sulfate respiration: proposal of the new genus “*Candidatus sulfobium*”. *Appl. Environ. Microbiol.* 84:e02224-17. 10.1128/AEM.02224-17 29247059 PMC5812927

