# Supplementary material for: Unraveling Nitrogen, Sulfur, and Carbon Metabolic Pathways and Microbial Community Transcriptional Responses to Substrate Deprivation and Toxicity Stresses in a Bioreactor Mimicking Anoxic Brackish Coastal Sediment Conditions
Source: Front Microbiol. 2022 Feb 23;13:798906. doi: 10.3389/fmicb.2022.798906 (PMC8906906; doi:10.3389/fmicb.2022.798906)
Supplement: Supplementary file 1 [file Data_Sheet_1.pdf]

Supplementary Material

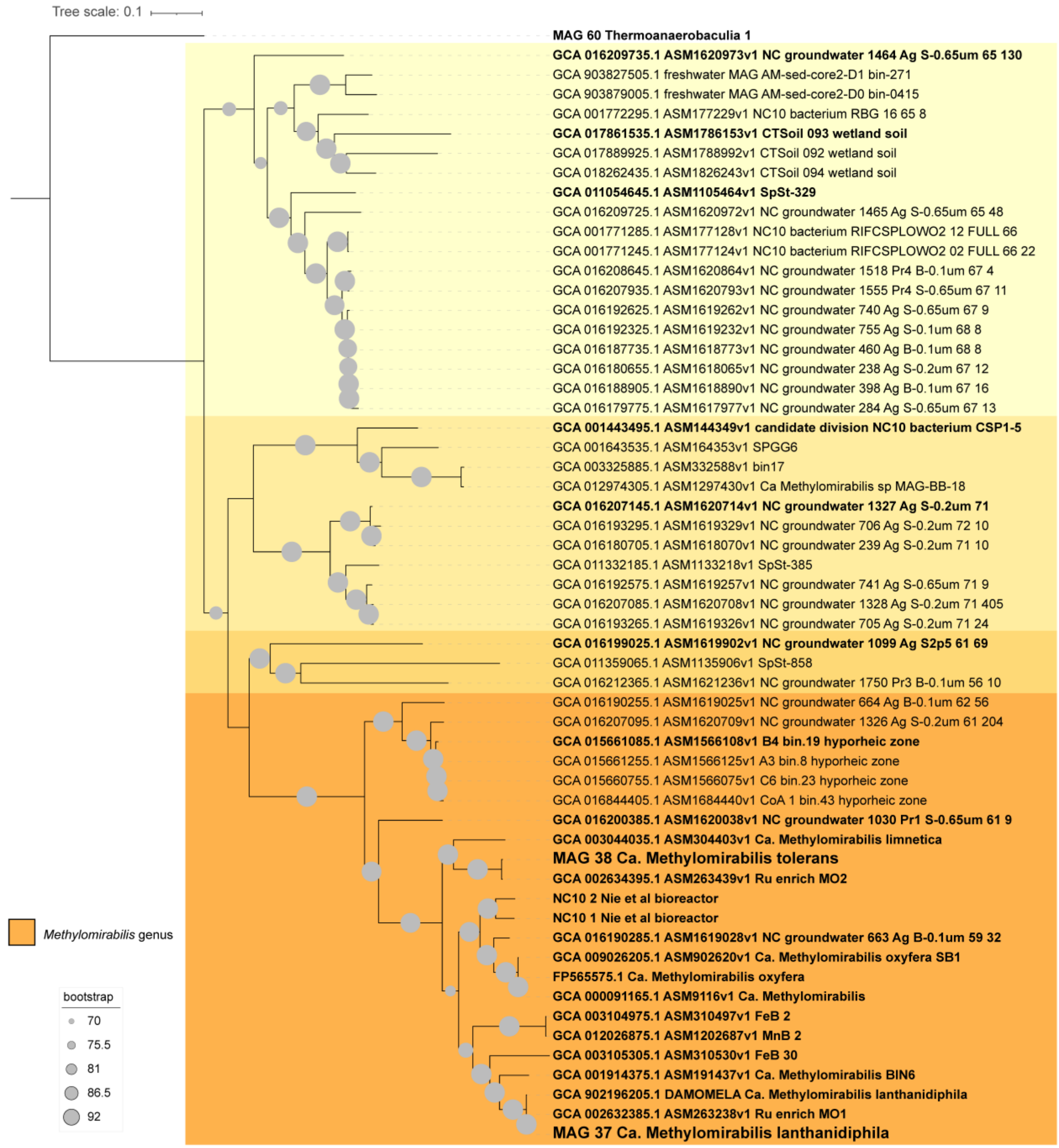

Supplementary Figure 1. Phylogenetic tree of NC10 phylum-affiliated genomes retrieved from NCBI (accession numbers are indicated), from Nie et al upon request (12), and from this study (MAG 37 and 38). Ninety-two genes were extracted with UBCG, concatenated and aligned with FastTree. The *candidatus* genus Methylomirabilis is highlighted in orange. Genomes in bold were used for average amino acid identity analyses in Supplementary Figure 2.

	GCA 016207145.1 ASM1620714v1 NC groundwater 1327 Ag S−0.2um 71 167	GCA 001443495.1 ASM144349v1 candidate division NC10 bacterium CSP1−5	GCA 016199025.1 ASM1619902v1 NC groundwater 1099 Ag S2p5 61 69	GCA 016209735.1 ASM1620973v1 NC groundwater 1464 Ag S−0.65um 65 130	GCA 011054645.1 ASM1105464v1 SpSt−329	GCA 017861535.1 ASM1786153v1 CTSoil 093 wetland soil	MAG 60 Thermoanaerobaculia 1	**MAG 38 Ca. Methylomirabilis tolerans**	GCA 002634395.1 ASM263439v1 Ru enrich MO2	GCA 003044035.1 ASM304403v1 Ca. Methylomirabilis limnetica	NC10 2 Nie et al bioreactor	NC10 1 Nie et al bioreactor	GCA 016190285.1 ASM1619028v1 NC groundwater 663 Ag B−0.1um 59 32	GCA 000091165.1 ASM9116v1 Ca. Methylomirabilis oxyfera	FP565575.1 Ca Methylomirabilis oxyfera	GCA 009026205.1 ASM902620v1 Ca Methylomirabilis oxyfera SB1	**MAG 37 Ca. Methylomirabilis lanthanidiphila**	GCA 902196205.1 DAMOMELA Ca Methylomirabilis lanthanidiphila	GCA 002632385.1 ASM263238v1 Ru enrich MO1	GCA 001914375.1 ASM191437v1 Ca. Methylomirabilis BIN6	GCA 003105305.1 ASM310530v1 FeB 30	GCA 012026875.1 ASM1202687v1 MnB 2	GCA 003104975.1 ASM310497v1 FeB 2	GCA 016200385.1 ASM1620038v1 NC groundwater 1030 Pr1 S−0.65um 61 9	GCA 015661085.1 ASM1566108v1 B4 bin.19 hyporheic zone
GCA 016207145.1 ASM1620714v1 NC groundwater 1327 Ag S−0.2um 71 167	100	55	52	50	55	47	42	51	51	51	44	51	51	51	51	51	51	51	51	48	50	51	52	53	53
GCA 001443495.1 ASM144349v1 candidate division NC10 bacterium CSP1−5	55	100	53	50	53	46	42	52	52	53	48	52	53	53	53	52	52	52	52	51	52	52	52	54	56
GCA 016199025.1 ASM1619902v1 NC groundwater 1099 Ag S2p5 61 69	52	53	100	48	52	46	40	53	53	52	46	52	53	53	53	53	53	53	53	51	51	52	53	54	55
GCA 016209735.1 ASM1620973v1 NC groundwater 1464 Ag S−0.65um 65 130	50	50	48	100	63	56	41	50	50	50	40	49	49	50	50	50	50	50	50	48	49	50	50	50	51
GCA 011054645.1 ASM1105464v1 SpSt−329	55	53	52	63	100	59	42	53	53	53	47	52	53	53	53	53	53	53	53	51	52	53	53	55	56
GCA 017861535.1 ASM1786153v1 CTSoil 093 wetland soil	47	46	46	56	59	100	40	47	46	46	41	46	46	46	46	46	47	47	47	45	46	46	46	46	47
MAG 60 Thermoanaerobaculia 1	42	42	40	41	42	40	100	42	41	41	39	41	42	41	41	41	42	42	42	41	41	42	42	41	42
**MAG 38 Ca. Methylomirabilis tolerans**	51	52	53	50	53	47	42	100	98	81	78	80	81	82	82	82	79	79	80	74	76	77	77	69	71
GCA 002634395.1 ASM263439v1 Ru enrich MO2	51	52	53	50	53	46	41	98	100	80	81	79	81	83	83	82	79	79	79	75	76	77	77	69	71
GCA 003044035.1 ASM304403v1 Ca. Methylomirabilis limnetica	51	53	52	50	53	46	41	81	80	100	72	77	80	80	80	79	77	77	77	73	76	76	76	70	72
NC10 2 Nie et al bioreactor	44	48	46	40	47	41	39	78	81	72	100	53	75	78	78	78	76	76	76	64	68	73	74	61	58
NC10 1 Nie et al bioreactor	51	52	52	49	52	46	41	80	79	77	53	100	84	84	84	84	80	80	80	75	78	78	79	68	70
GCA 016190285.1 ASM1619028v1 NC groundwater 663 Ag B−0.1um 59 32	51	53	53	49	53	46	42	81	81	80	75	84	100	86	86	86	81	81	81	77	78	80	80	70	71
GCA 000091165.1 ASM9116v1 Ca. Methylomirabilis oxyfera	51	53	53	50	53	46	41	82	83	80	78	84	86	100	100	100	81	81	82	77	79	79	79	69	70
FP565575.1 Ca. Methylomirabilis oxyfera	51	53	53	50	53	46	41	82	83	80	78	84	86	100	100	100	81	81	82	77	79	79	79	69	70
GCA 009026205.1 ASM902620v1 Ca. Methylomirabilis oxyfera SB1	51	52	53	50	53	46	41	82	82	79	78	84	86	100	100	100	81	81	81	77	79	79	79	69	71
**MAG 37 Ca. Methylomirabilis lanthanidiphila**	51	52	53	50	53	47	42	79	79	77	76	80	81	81	81	81	100	100	100	82	81	80	80	69	71
GCA 902196205.1 DAMOMELA Ca. Methylomirabilis lanthanidiphila	51	52	53	50	53	47	42	79	79	77	76	80	81	81	81	81	100	100	100	82	81	80	80	69	71
GCA 002632385.1 ASM263238v1 Ru enrich MO1	51	52	53	50	53	47	42	80	79	77	76	80	81	82	82	81	100	100	100	82	81	80	80	69	71
GCA 001914375.1 ASM191437v1 Ca. Methylomirabilis BIN6	48	51	51	48	51	45	41	74	75	73	64	75	77	77	77	77	82	82	82	100	78	77	78	64	65
GCA 003105305.1 ASM310530v1 FeB 30	50	52	51	49	52	46	41	76	76	76	68	78	78	79	79	79	81	81	81	78	100	78	79	67	69
GCA 012026875.1 ASM1202687v1 MnB 2	51	52	52	50	53	46	42	77	77	76	73	78	80	79	79	79	80	80	80	77	78	100	100	68	70
GCA 003104975.1 ASM310497v1 FeB 2	52	52	53	50	53	46	42	77	77	76	74	79	80	79	79	79	80	80	80	78	79	100	100	68	70
GCA 016200385.1 ASM1620038v1 NC groundwater 1030 Pr1 S−0.65um 61 9	53	54	54	50	55	46	41	69	69	70	61	68	70	69	69	69	69	69	69	64	67	68	68	100	77
GCA 015661085.1 ASM1566108v1 B4 bin.19 hyporheic zone	53	56	55	51	56	47	42	71	71	72	58	70	71	70	70	71	71	71	71	65	69	70	70	77	100

Genus *Methylomirabilis*

same species

Supplementary Figure 2. Average amino acid identity (AAI) matrix highlighting the *candidatus* genus Methylomirabilis (yellow) and some genomes of the same species (red).

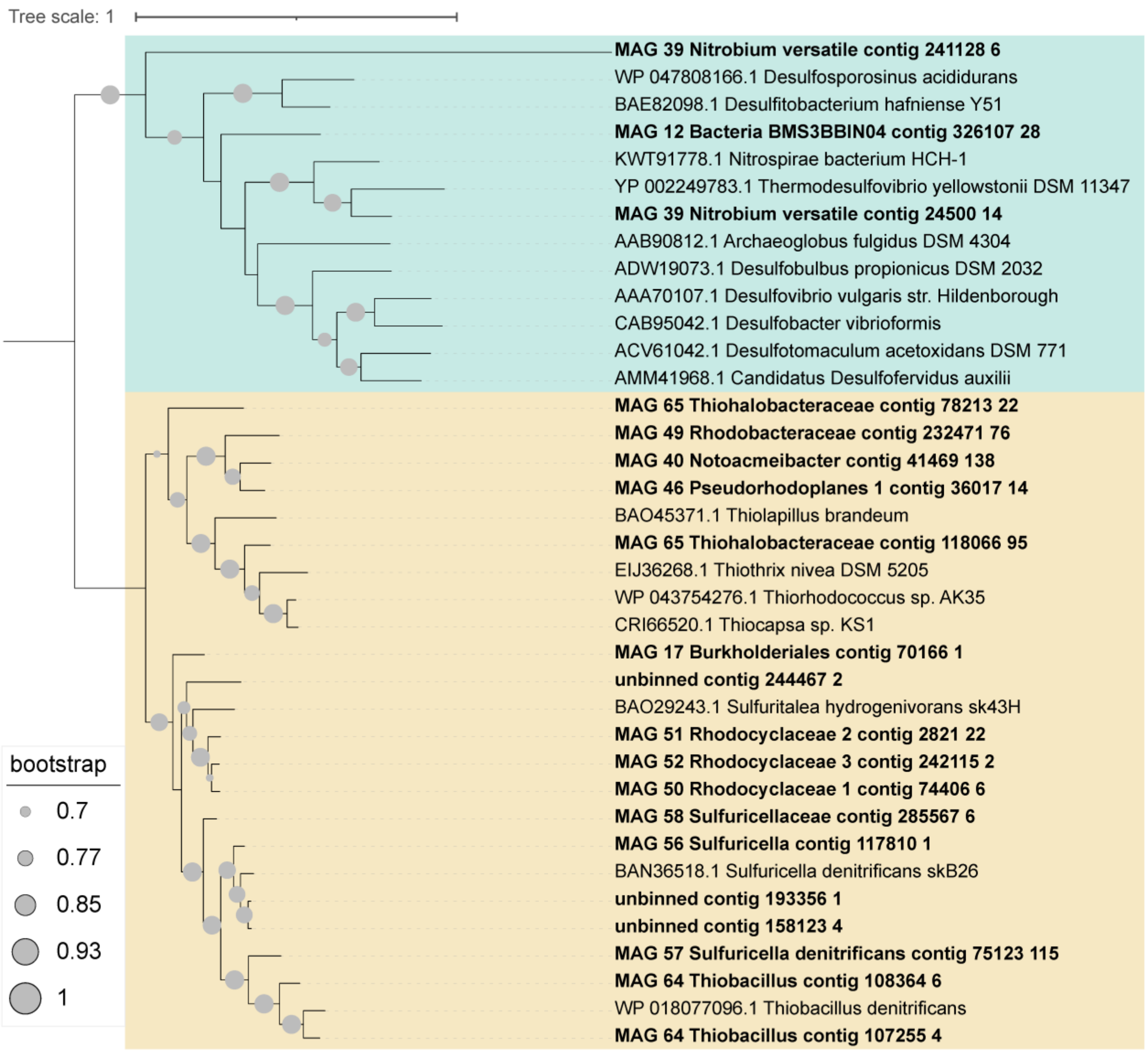

Supplementary Figure 3. Phylogenetic tree of DsrA sequences from this study (in bold) and reference sequences (indicated by NCBI accession numbers). Sequences putatively assigned to the sulfur reduction direction are highlighted in blue, and to the oxidative direction are highlighted in yellow.

Supplementary Table 1. Specific dates, duration (left) and numbering (right) of the ammonium removal and toxicity stresses experiments. Right columns- specific biomass collection dates and numbering for metagenomics (G), metatranscriptomics (T), and RT-qPCR (R) before/after substrate removal and stresses. (dd.mm.yyyy).

**Experiment date/ duration**	**metaG (G)**		**metaT (T)**		**RT-qPCR (R)**	
22.6.2016	**Combination of enrichment cultures (Reactor started)**					
22.6.2016 - 11.03.2017 ~ 1 year	**0 No changes – stable condition**					
	02.01.2017	**G0***	01.02.2017	**T0**	01.02.2017	**R0**
12.03.2017-04.04.2017 ~ 7 weeks	**1 Ammonium removal (7-0mM)**					
18.04.2017-20.06.2017 ~ 10 weeks			18.04.2017	**T1**		
			20.06.2017	**T2**	01.08.2017	**R1**
29.10.2017-01.10.2019 ~ 2 years	**2 Ammonium restored (7mM) – stable condition**					
	07.11.2018	**G1***				
	19.02.2019	**G2**	17.12.2018	**T3**		
01.10.2019-14.10.2019 ~ 15 days	**3 Methane saturation removed**					
	25.11.2019	**G3**	26.11.2019	**T4**	26.11.2019	**R3**
24.11.2019-02.02.2020 ~ 10 weeks	**4 Methane saturation restored and external nitric oxide supply (~1-5%)**					
	06.02.2020	**G4**				
06.02.2020-02.04.2020 ~ 7 weeks	**5 Only sulfide (6.7mM) and further increased external nitric oxide supply (~10-13%)**					
	02.04.2020	**G5**	02.04.2020	**T5**	02.04.2020	**R4**

*DNA extractions (DNAe) not included in analysis but used for assembly improvement (total of 8 metagenomes)

G0.1: using CTAB, alternative DNAe method to Power Soil

G1.1: DNAe from bioreactor wall to improve assembly

Supplementary Table 2. Transcript per million (TPM) values for each gene and genome in this study (excel spreadsheet).

Supplementary Table 3. RT-qPCR R0-R1 and R3-R3 results calculated in $2^{\wedge}\Delta\Delta CT$ values for selected functional genes using three biological and technical duplicates per time point (R).

**$2^{\wedge}(-\Delta\Delta CT)$**	***hzsA***	***pmoA***	***mcrA***
**R0-R1**	0.00	1.24	1.15
**R3-R4**	0.02	0.00	0.00

Supplementary Table 4. Gene annotations and loci for MAGs included in Figure 4 (excel spreadsheet).
